# Genetic Causes and Genomic Consequences of Breakdown of Distyly in *Linum trigynum*

**DOI:** 10.1093/molbev/msae087

**Published:** 2024-05-06

**Authors:** Juanita Gutiérrez-Valencia, Panagiotis-Ioannis Zervakis, Zoé Postel, Marco Fracassetti, Aleksandra Losvik, Sara Mehrabi, Ignas Bunikis, Lucile Soler, P William Hughes, Aurélie Désamoré, Benjamin Laenen, Mohamed Abdelaziz, Olga Vinnere Pettersson, Juan Arroyo, Tanja Slotte

**Affiliations:** Department of Ecology, Environment and Plant Sciences, Science for Life Laboratory, Stockholm University, Stockholm, Sweden; Department of Ecology, Environment and Plant Sciences, Science for Life Laboratory, Stockholm University, Stockholm, Sweden; Department of Ecology, Environment and Plant Sciences, Science for Life Laboratory, Stockholm University, Stockholm, Sweden; Department of Ecology, Environment and Plant Sciences, Science for Life Laboratory, Stockholm University, Stockholm, Sweden; Department of Ecology, Environment and Plant Sciences, Science for Life Laboratory, Stockholm University, Stockholm, Sweden; Department of Ecology, Environment and Plant Sciences, Science for Life Laboratory, Stockholm University, Stockholm, Sweden; Department of Immunology, Genetics and Pathology, Uppsala Genome Center, Science for Life Laboratory, Uppsala University, Uppsala, Sweden; Department of Medical Biochemistry and Microbiology, Uppsala University, National Bioinformatics Infrastructure Sweden (NBIS), Science for Life Laboratory, Uppsala University, Uppsala, Sweden; Department of Ecology, Environment and Plant Sciences, Science for Life Laboratory, Stockholm University, Stockholm, Sweden; Department of Ecology, Environment and Plant Sciences, Science for Life Laboratory, Stockholm University, Stockholm, Sweden; Department of Ecology, Environment and Plant Sciences, Science for Life Laboratory, Stockholm University, Stockholm, Sweden; Department of Genetics, University of Granada, Granada, Spain; Department of Immunology, Genetics and Pathology, Uppsala Genome Center, Science for Life Laboratory, Uppsala University, Uppsala, Sweden; Department of Plant Biology and Ecology, University of Seville, Seville, Spain; Department of Ecology, Environment and Plant Sciences, Science for Life Laboratory, Stockholm University, Stockholm, Sweden

**Keywords:** homostyly, self-fertilization, distribution of fitness effects, genome assembly, plant mating system

## Abstract

Distyly is an iconic floral polymorphism governed by a supergene, which promotes efficient pollen transfer and outcrossing through reciprocal differences in the position of sexual organs in flowers, often coupled with heteromorphic self-incompatibility. Distyly has evolved convergently in multiple flowering plant lineages, but has also broken down repeatedly, often resulting in homostylous, self-compatible populations with elevated rates of self-fertilization. Here, we aimed to study the genetic causes and genomic consequences of the shift to homostyly in *Linum trigynum*, which is closely related to distylous *Linum tenue.* Building on a high-quality genome assembly, we show that *L. trigynum* harbors a genomic region homologous to the dominant haplotype of the distyly supergene conferring long stamens and short styles in *L. tenue*, suggesting that loss of distyly first occurred in a short-styled individual. In contrast to homostylous *Primula* and *Fagopyrum*, *L. trigynum* harbors no fixed loss-of-function mutations in coding sequences of *S-*linked distyly candidate genes. Instead, floral gene expression analyses and controlled crosses suggest that mutations downregulating the *S-*linked *LtWDR-44* candidate gene for male self-incompatibility and/or anther height could underlie homostyly and self-compatibility in *L. trigynum*. Population genomic analyses of 224 whole-genome sequences further demonstrate that *L. trigynum* is highly self-fertilizing, exhibits significantly lower genetic diversity genome-wide, and is experiencing relaxed purifying selection and less frequent positive selection on nonsynonymous mutations relative to *L. tenue*. Our analyses shed light on the loss of distyly in *L. trigynum*, and advance our understanding of a common evolutionary transition in flowering plants.

## Introduction

Flowering plants exhibit a remarkable diversity of floral and reproductive structures, in large part due to selection for increased pollination and reproductive efficiency (reviewed in Schiestl and Johnson 2013; Simón-Porcar et al. 2024). Understanding adaptation to different pollination modes is important, because this process can contribute to speciation, and is likely to underlie repeated convergent evolution of floral traits in flowering plants (reviewed in Barrett 2010; Ashworth et al. 2015). Evolutionary biologists have therefore long been fascinated by floral adaptations to pollination modes (Darwin 1876, 1877).

**Fig. 1.**
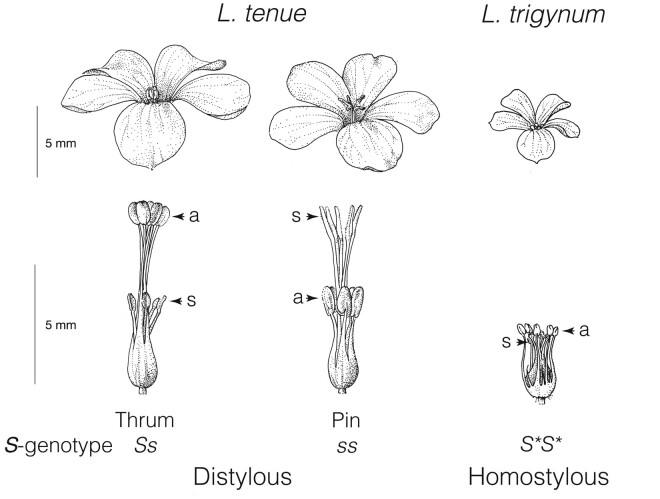
Flower morphs of the distylous *L. tenue* and the homostylous *L. trigynum*. The expected genotypes of thrum (left), pin (middle), and homostylous (right) flowers are indicated. In thrum flowers, pollen-producing anthers (indicated by a) are positioned above the short styles with receptive stigmas (indicated by s) in a low position in the flower, while pin individuals show the opposite reciprocal arrangement. Homostylous flowers show reduced herkogamy (i.e. anthers and stigmas in closer proximity). *S* = dominant haplotype, *s* = recessive haplotype, and S* = haplotype homologous to the *L. tenue S* allele. The position of stigmas and anthers are indicated in each morph. Illustrations by Alison Cutts.

One such adaptation is distyly, a floral polymorphism that promotes efficient pollen transfer and outcrossing via insect pollinators (Darwin 1877; Lloyd and Webb 1992; Simón-Porcar et al. 2024, reviewed by Barrett 2019). Natural populations of distylous species are polymorphic for floral morph, such that individuals have one of two types of flowers that differ reciprocally in the positions of male and female sexual organs (anthers and stigmas, respectively) within the flower. Pin individuals have long styles (L-morph) and short stamens with stigmas at a high position and anthers at a low position in the flower, whereas thrum individuals have the reciprocal arrangement with short styles (S-morph) and long stamens (Fig. 1). In most distylous species, these morphological differences are coupled with a heteromorphic self-incompatibility (SI) system which allows inbreeding avoidance (Charlesworth and Charlesworth 1979b) and reinforces disassortative mating by preventing self- and intra-morph fertilization.

Despite its multitrait nature, distyly is inherited as a single Mendelian locus, called the distyly supergene or the *S-*locus, which governs both morphological differences and heteromorphic SI. Early work suggested that the distyly supergene typically harbors two alleles: one dominant exclusive to thrums and one recessive for which pins are homozygous (Bateson and Gregory 1905; Laibach 1923;  Fig. 1). It is only recently that distyly supergenes have begun to be sequenced and characterized in detail (reviewed in Gutiérrez-Valencia et al. 2021). So far, characterization of independently evolved distyly supergenes in six systems (*Fagopyrum*: Yasui et al. 2012; Fawcett et al. 2023; *Primula*: Li et al. 2016; *Turnera*: Shore et al. 2019; *Linum*: Gutiérrez-Valencia et al. 2022a; *Nymphoides indica*: Yang et al. 2023; *Gelsemium*: Zhao et al. 2023) suggest a remarkable degree of convergence in the genetic architecture of distyly supergenes. Common features of independently evolved distyly supergenes include a genetic architecture featuring length differences between the dominant and recessive supergene haplotypes, resulting in hemizygosity in thrum individuals and thrum-specific expression of *S-*linked genes (Gutiérrez-Valencia et al. 2022a). In addition, elevated repeat content is frequently found at distyly supergenes (Gutiérrez-Valencia et al. 2022a).

Style length polymorphisms, including distyly, have evolved independently multiple times in independent flowering plant lineages (Naiki 2012; Simón-Porcar et al. 2024). However, distyly has also broken down frequently, often resulting in homostylous species that are monomorphic and self-compatible (SC) with anthers and stigma at the same level (nonherkogamous; reviewed by Ganders 1979; note that here we restrict the use of the term homostylous to derived, monomorphic populations or species with anthers and stigmas at the same height, as in Darwin (1877)). Given their capacity for self-fertilization, facilitated by the joint occurrence of reduced herkogamy and SC, homostylous individuals (homostyles) can be favored whenever insect-mediated pollination and fertilization become unreliable, due to selection for reproductive assurance (e.g. Piper et al. 1986; Yuan et al. 2017). Genomic characterization of transitions from distyly to homostyly may therefore allow the identification of genetic changes underlying this mating system shift, as well as its population genomic consequences.

Because early models posited that thrum plants were heterozygous at the distyly supergene (Ernst 1936), it was frequently hypothesized that rare recombination events between dominant and recessive *S*-haplotypes caused homostyly (Dowrick 1956; Charlesworth and Charlesworth 1979a, reviewed by Ganders 1979). This idea has been revisited after the realization that thrums are predominantly hemizygous rather than heterozygous at the distyly *S*-locus in several distylous systems (Li et al. 2016; Shore et al. 2019; Gutiérrez-Valencia et al. 2022a; Fawcett et al. 2023). Since hemizygosity precludes the possibility of recombination between the dominant and recessive haplotypes, other genetic causes of distyly breakdown should be considered. First, genetic modifiers not linked to the distyly supergene could act by reducing floral herkogamy (Ganders 1979; Mather and De Winton 1941). Second, homostyly could evolve as a consequence of loss-of-function mutations at *S*-linked genes (as suggested by Ernst 1936). Such mutations could result in either long homostyles, with both anthers and stigma in a high position in the flower, or short homostyles, with their sexual organs in a low position in the flower.

Theory predicts that long homostyles, which exhibit functional dominant male SI in combination with recessive style length and female SI function should be favored during the establishment of homostyly, as they produce only homostylous offspring when pollinating compatible pin plants, in contrast to short homostyles which produce 50% homostylous offspring in crosses to compatible thrum plants (Dowrick 1956; Charlesworth and Charlesworth 1979a). As a consequence, long homostyles will spread faster than short homostyles at the initial stages of evolution of homostyly from distyly (although short homostyles can fix in the absence of long homostyles; Dowrick 1956; Charlesworth and Charlesworth 1979a).

In systems with hemizygous *S*-loci, it seems likely that homostyly would arise by mutation and not recombination. So far, results on the breakdown of distyly in *Primula* and *Fagopyrum* are in line with this prediction. In both systems, mutations affecting *S-*linked genes responsible for female SI function and short styles (i.e. thrum-exclusive *CYP734A50* in *Primula* encoding a brassinosteroid-inactivating enzyme, and *S-ELF3* in *Fagopyrum*) can readily lead to the formation of long-homostylous SC plants, because these *S*-linked genes jointly govern style length and female SI (Huu et al. 2016, 2022; Fawcett et al. 2023). Moreover, independently evolved homostyles in natural populations of *Primula vulgaris* harbor putative loss-of-function mutations in *CYP734A50* (Mora-Carrera et al. 2023). Mutations at *S*-linked genes, particularly in genes affecting style length and female SI, thus constitute a feasible pathway to the evolution of homostyly in natural populations, but it remains unknown if similar events have unfolded in lineages of distylous plants other than *Primula* and *Fagopyrum*.

Genomic studies hold the promise to elucidate potential genetic causes of loss of distyly and to quantify the impact of homostyly on outcrossing rates, as well as to characterize the consequences for patterns of polymorphism and the efficacy of selection. If the evolution of homostyly is associated with shifts to high selfing rates, we expect the transition to result in marked reductions in the effective population size (*N*_e_), exacerbated by linked selection due to reduced effective recombination rates in selfers, and potentially by founder events and bottlenecks associated with selfing (reviewed by Wright et al. 2013; Slotte 2014; Hartfield et al. 2017; Cutter 2019). In combination, these processes should result in reduced genetic diversity genome-wide in selfing homostylous species compared with their distylous relatives, which can also lead to more marked population structure and a decreased efficacy of selection, especially against weakly deleterious mutations but possibly also a reduced efficacy of positive selection. Although transitions from distyly to homostyly have occurred repeatedly in the history of flowering plants, the genomic consequences of this transition have so far primarily been studied in one system, *Primula* (e.g. Zhong et al. 2019; Wang et al. 2021).

*Linum* is a promising system for studying the evolution and breakdown of distyly (e.g. Armbruster et al. 2006; McDill et al. 2009; Gutiérrez-Valencia et al. 2022a; Innes et al. 2023), because it shows a remarkable diversity of stylar conditions, including several independent losses of distyly (Ruiz-Martín et al. 2018; Maguilla et al. 2021). In agreement with the expectation that selfers have improved colonization ability (Baker 1955; Stebbins 1957), phylogenetic analyses suggest that the evolution of homostyly is associated with the expansion of *Linum* outside its center of origin in the Western Palearctic (Maguilla et al. 2021). It remains largely unknown how homostyly has evolved in *Linum*, but we have recently shown that, similar to other distylous lineages, the distyly *S*-locus in *Linum tenue* harbors a hemizygous region exclusively inherited by thrums (Gutiérrez-Valencia et al. 2022a). Importantly, this hemizygous region harbors a candidate gene for style length (*LtTSS1*), and the gene *LtWDR-44*, likely involved in anther elongation and/or pollen functioning (discussed in Gutiérrez-Valencia et al. 2022a). If mutations that impair *LtTSS1* function confer recessive style length and female SI function, we would expect such mutations to be favored during evolution of homostyly (Dowrick 1956; Charlesworth and Charlesworth 1979a).

Here, we investigated the genetic causes and evolutionary genomic consequences of loss of distyly in *Linum trigynum*, an annual SC species distributed across the Mediterranean Basin and Western Asia (POWO 2024), in which homostyly is a derived state (Ruiz-Martín et al. 2018). For this purpose, we assembled and annotated a high-quality genome sequence of the homostylous *L. trigynum* using PacBio high-fidelity (HiFi) long reads and chromatin conformation capture (Hi-C) data. While a high-quality genome assembly is available for the closely related distylous *L. tenue* (Gutiérrez-Valencia et al. 2022a), no reference genomes of closely related homostylous *Linum* species were previously available. The new genomic resources for *L. trigynum* that we present here enable in-depth investigation of the evolution of homostyly and its genomic consequences.

To test hypotheses on the genetic basis of loss of distyly, we compared our *L. trigynum* genome assembly as well as two additional linked-read draft assemblies of *L. trigynum* to eight genome assemblies of the closely related distylous species *L. tenue* (Gutiérrez-Valencia et al. 2022a) to search for mutations at *S*-linked genes. We also made use of expression data to investigate patterns of differential expression at genes within the distyly supergene. To characterize the timing of the split between these species, we analyzed polymorphism data based on 224 whole-genome sequences from eight natural populations each of *L. trigynum* and *L. tenue*. Finally, we conducted comparative population genomic analyses to test whether the shift to homostyly in *L. trigynum* was associated with reduced nucleotide diversity genome-wide, elevated inbreeding levels, increased population structure and a reduced efficacy of both purifying and positive selection, as predicted if the shift led to elevated selfing rates.

## Results

### A Chromosome-Scale Genome Assembly of *L. trigynum*

**Fig. 2.**
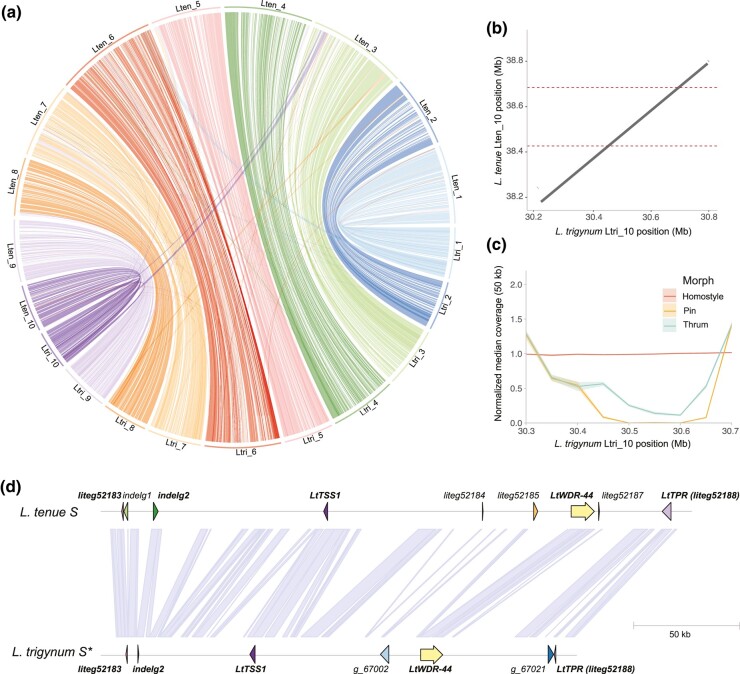
Broad-scale synteny between *L. trigynum* and *L. tenue* and comparative analyses of the *S-*locus region. a) Circos plot showing broad-scale synteny between *L. tenue* (top) and *L. trigynum* (bottom). b) Dot plot showing significant minimap2 alignments between the genomic region harboring the dominant *S-*haplotype of *L. tenue* and a region of *L. trigynum* chromosome 10. The limits of the *L. tenue S-*locus are indicated by dotted lines. c) Plot of median and 95% confidence intervals of median coverage in 50 kb windows for *L. trigynum* (homostyle), *L. tenue* thrum and pin individuals, all mapped to our *L. trigynum* assembly. d) Comparison of gene content at the *S-*haplotype of *L. tenue* (including genes *liteg52183* and *liteg52188* not within the hemizygous region of the *S-*locus; entire length of *S-*locus ∼261 kb) and corresponding region (here named S*; length ∼208 kb) in *L. trigynum*. Names of genes shared between the *L. tenue S-*haplotype and the L. trigynum S*-haplotype are written in bold text, and lines connecting the two haplotypes indicate orthologous regions.

To provide a genomic framework for studies of the evolution of homostyly in *L. trigynum*, we generated a high-quality *L. trigynum* genome assembly based on HiFi PacBio long reads (∼41 × coverage) and Hi-C data from a single homostylous *L. trigynum* individual (see Materials and Methods and supplementary table S1 and information, Supplementary Material online for sample information). The resulting assembly was highly complete (complete BUSCOs [Benchmarking Universal Single-Copy Orthologs] = 96.6%; supplementary table S2 and information, Supplementary Material online) and spanned 498 Mb (N50 = 47.03 Mb), divided in ten pseudochromosomes and five scaffolds (<500 kb) with large-scale synteny to the genome assembly of distylous *L. tenue* (Gutiérrez-Valencia et al. 2022a; Fig. 2a; supplementary fig. S1 and information, Supplementary Material online). Our chromosome-scale assembly has the same number of pseudochromosomes as the haploid chromosome number in *L. trigynum* and *L. tenue* (*n =* 10 for both diploid annual species; Valdés-Florido et al. 2023). Repetitive sequences constituted 56.49% of the assembly, and we annotated a total of 54,692 coding genes, similar to the number in *L. tenue* (Gutiérrez-Valencia et al. 2022a).

We supplemented our PacBio genome assemblies of *L. trigynum* and *L. tenue* with two additional draft assemblies of *L. trigynum* based on 10× Chromium linked-read sequencing data (contig N50 = 4.82 vs. 4.47 Mb; assembly size = 461.69 vs. 463.46 Mb), and seven additional *L. tenue* linked-read assemblies for thrum individuals which carry both the dominant and recessive haplotypes of the *S*-locus (supplementary table S3 and information, Supplementary Material online).

### Analyses of the *S*-locus Region Elucidate the Origin of Homostyly

Whole-genome alignments of our high-quality *L. trigynum* and *L. tenue* assemblies showed that *L. trigynum* carries a region on chromosome 10 that is homologous to the longer, dominant allele of the *L. tenue* distyly *S*-locus (Fig. 2b). In contrast to *L. tenue*, which is polymorphic for a ∼260 kb indel at the *S-*locus (Gutiérrez-Valencia et al. 2022a), analyses of linked-read assemblies and genome coverage at this region based on short-read data from 104 *L. trigynum* individuals suggest that no such presence–absence variation is present in *L. trigynum*. Instead, all *L. trigynum* individuals harbor a haplotype similar to the longer *S*-haplotype that is dominant in *L. tenue* (Fig. 2c). This result suggests that events affecting the functioning of the supergene in thrum individuals led to the breakdown of distyly, and that the haplotype identified in *L. trigynum* is likely derived from the dominant *S-*haplotype.

We then investigated the gene content in the *L. trigynum* genome region homologous to the dominant *S-*haplotype of *L. tenue*. After curation of *S*-locus annotation (see Materials and Methods for details), we retained nine *S*-linked genes (Fig. 2d) in *L. tenue*. Out of these, five homologous *S-*linked genes were present in our high-quality long-read *L. trigynum* assembly (Fig. 2d). These included the thrum-specific gene *LtTSS1* which has pistil-specific expression and is likely to reduce cell length in thrum styles, and *LtWDR-44* potentially involved in controlling anther position and/or pollen incompatibility in *L. tenue* (discussed in Gutiérrez-Valencia et al. 2022a). In addition, *L. trigynum* harbored homologs of genes at the 5′ and 3′ ends of the *S-*locus region (*liteg52183* and *LtTPR*, respectively, Fig. 2d). The remaining genes, which are of unknown function (Gutiérrez-Valencia et al. 2022a), were not present in the *L. trigynum* genome assembly (Fig. 2d). Importantly, haplotype network analyses of *LtTSS1* and *LtWDR-44* indicate that variation at the *L. trigynum S-*locus stems from a single *S-*haplotype from its distylous ancestor (supplementary fig. S2 and information, Supplementary Material online), and they further support the conclusion that loss of distyly first occurred in a short-styled individual, as suggested by the coverage-based analyses.

Next, we compared sequences of *L. tenue* and *L. trigynum* to identify candidate loss-of-function changes at the *S-*locus region affecting gene function in *L. trigynum*, and to quantify synonymous and nonsynonymous divergence between *L trigynum* and the dominant *S-*haplotype of *L. tenue.* We only identified putative loss-of-function changes in *L. trigynum* in the gene *LtTSS1* (Table 1). This gene harbors one exon, as demonstrated by our updated annotation, validated by PCR assays of cDNA structure and similar to the gene structure of *Thrum Style Specific 1* (*TSS1*) in *Linum grandiflorum* (Ushijima et al. 2012; see Materials and Methods for details; supplementary fig. S3, table S4, methods, and information, Supplementary Material online). In *LtTSS1*, we identified a C-to-A mutation resulting in a premature stop codon in *L. trigynum*, that was located near the end of the predicted protein (eight codon positions prior to the regular stop codon), after the two conserved motifs previously identified in the *L. grandiflorum* homolog *TSS1* (Ushijima et al. 2012). This C-to-A mutation was not fixed in *L. trigynum* but segregated at a frequency of 0.26 in our population data set. Taken together, the genic location and population frequency of this mutation suggests that it is unlikely to cause loss of distyly. Although all genes also harbored nonsynonymous mutations, there were no frameshift or other major-effect mutations in the coding sequence of the other analyzed genes, nor markedly accelerated rates of nonsynonymous substitution (Table 1). Finally, we used our repeat annotations to compare the transposable element (TE) content in the promoter region (2 kb upstream of the transcription start site) of *LtTSS1* and *LtWDR-44* in *L. tenue* and *L. trigynum*. We found no differences in the TE content in the promoter region of *LtTSS1* (supplementary table S5 and information, Supplementary Material online), but the TE content in the promoter region of *LtWDR-44* differed between species. *Linum trigynum* harbored long terminal repeat (LTR)/Ty3-like LTR retrotransposon insertions in the *LtWDR-44* promoter that were not detected in *L. tenue*, and the type of DNA transposons detected also differed between the two species (supplementary table S5 and information, Supplementary Material online).

**Table 1:** Sequence divergence between *L. trigynum* and *L. tenue* genome assemblies at the five homologous genes they share in the *S-*locus region

Gene	*n_Ltri_* ^a^	*n_Lten_* ^b^	*d_S_*	SE*_dS_*	*d_N_*	SE*_dN_*	Coding sites	Major-effect
*LITEG00000052183*	3	4	0.10	0.034	0.018	0.007	176	0
*indelg2*	3	6	0.036	0.023	0.073	0.020	86	0
*LtTSS1*	3	8	0.021	0.015	0.006	0.004	144	Segregating premature stop
*LtWDR-44*	3	8	0.017	0.005	0.012	0.002	925	0
*LtTPR LITEG00000052188*	3	5	0.018	0.010	0.009	0.004	267	0

Synonymous and nonsynonymous substitution rates are indicated by *d_S_* and *d_N_*, respectively. Standard errors (SE) of both are given as well as the number of major-effect mutations.

^a^Sample size, *L. trigynum*.

^b^Sample size, *L. tenue*.

### The S-linked Gene *LtWDR-44* is Downregulated in Floral Buds of *L*. *trigynum*

**Fig. 3.**
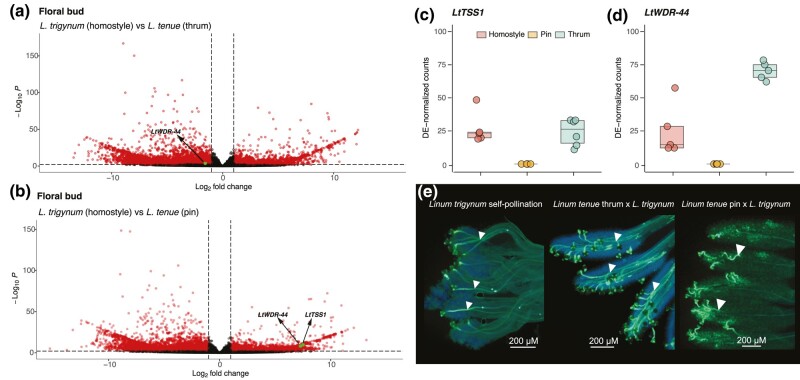
Differential expression of *S-*linked candidate genes in floral buds and pollination assays suggest a role for downregulation of *LtWDR-44* in transition to homostyly. a) Volcano plot depicting fold change versus significance of differential expression (log_2_-fold change *L. trigynum*/*L. tenue*) between *L. trigynum* homostyles and *L. tenue* thrums. Significant *S-*linked genes (only *LtWDR-44* in this analysis) are indicated. b) Volcano plot depicting fold change versus significance of differential expression (log_2_-fold change *L. trigynum*/*L. tenue*) between *L. trigynum* homostyles and *L. tenue* pins. Significant *S-*linked genes (*LtWDR-44* and *LtTSS1*) are indicated. c) Normalized counts for *LtTSS1* in *L. trigynum* homostyles, *L. tenue* pins and *L. tenue* thrums. d) Normalized counts for *LtWDR-44* in *L. trigynum* homostyles, *L. tenue* pins, and *L. tenue* thrums. e) Representative epifluorescence micrographs of pollination assays demonstrating self-compatibility of *L. trigynum* (left), a compatible reaction when pollinating *L. tenue* thrum (middle) but not pin (right) with *L. trigynum* pollen. Pollen tubes are indicated by arrows. Note that the site of pollen tube rejection is in the style in *Linum*, such that an incompatible cross yields shorter, aborted pollen tubes. Scale bars indicate the degree of magnification.

To determine if distyly breakdown in *L. trigynum* was associated with changes in transcript abundance at candidate genes, we contrasted gene expression at *S*-linked genes in *L. tenue* thrums and *L. trigynum* homostyles. We focused on detecting altered expression of any of the genes shared between the dominant *S* allele of *L. tenue* and its derived allele fixed in *L. trigynum* (i.e. *liteg52183*, *indelg2*, *LtTSS1*, *LtWDR44*, and *LtTPR/liteg52188*). Out of these genes, only the stamen length and/or male SI candidate gene *LtWDR-44* was significantly differentially expressed in floral buds, being downregulated in floral buds of *L. trigynum* compared with *L. tenue* (Log_2_-fold change = −1.58, *P* < 0.01; Fig. 3). *LtWDR-44* was not differentially expressed between *L. tenue* thrums and *L. trigynum* homostyles in leaves (supplementary fig. S4 and information, Supplementary Material online). None of the remaining *S*-linked genes showed significantly different levels of expression in floral buds or leaves between *L. tenue* thrum and homostylous *L. trigynum* (supplementary fig. S4 and information, Supplementary Material online).

### Pollination Assays Indicate Altered Male SI in *L*. *trigynum*

The evolution of homostyly from distyly is often associated with a shift from heteromorphic SI to SC (reviewed by Barrett 2019). If the *S-*linked gene *LtWDR-44* is a determinant of male SI, as we previously hypothesized (Gutiérrez-Valencia et al. 2022a), then we would expect downregulation of this gene to affect the male (pollen) SI expressed by *L. trigynum*. Specifically, we would expect *L. trigynum* pollen to behave similarly to *L. tenue* pin pollen and elongate successfully in thrum but not pin *L. tenue* pistils. Crossing assays followed by pollen tube staining showed that *L. trigynum* grew long pollen tubes in pistils of *L. tenue* thrum plants, but not in pistils of *L. tenue* pin plants (Fig. 3e; supplementary fig. S5 and information, Supplementary Material online; note that for incompatible crosses, pollen tube rejection occurs in variable sites along the style in *Linum*, but mostly in the upper part; Murray 1986). These results are consistent with expectations if downregulation of *LtWDR-44* in *L. trigynum* alters male SI function. A switch in male incompatibility phenotype from thrum to pin-type in an individual with functional thrum-type female incompatibility reaction would be expected to lead to SC.

### High Levels of Inbreeding in the Homostylous *L*. *trigynum*

**Fig. 4.**
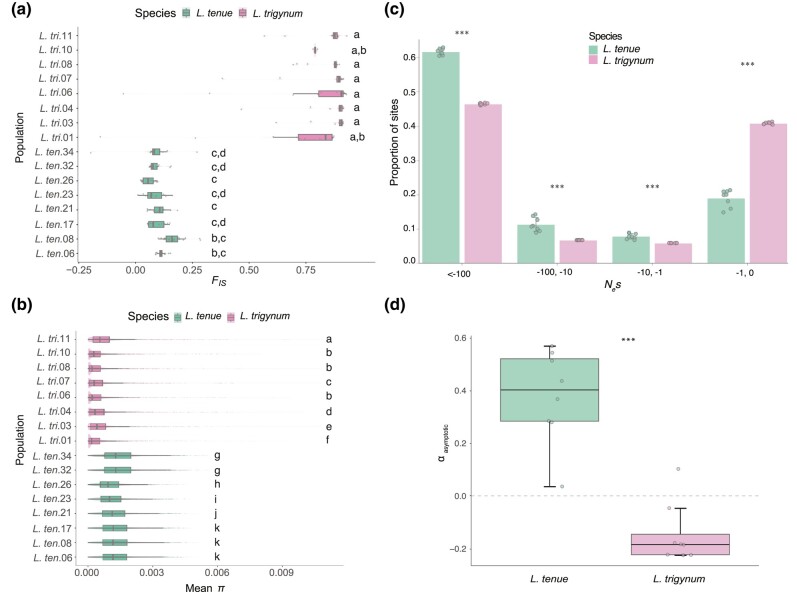
The shift to homostyly in *L. trigynum* is associated with higher levels of inbreeding, reduced genome-wide polymorphism, and a reduced efficacy of purifying and positive selection. a) Inbreeding coefficient (*F*_IS_) estimates for *L. trigynum* and *L. tenue* indicates higher inbreeding in *L. trigynum.* Boxplots show the distribution of *F*_IS_ estimates separately for each of eight *L. trigynum* populations (marked with a *L. tri.* prefix and a population number) and for each of eight *L. tenue* populations (marked with a *L. ten.* prefix and a population number, see supplementary table S1, Supplementary Material online). b) Comparisons of *π* estimates in windows of 100 kb across the genome show drastically lower nucleotide diversity in *L. trigynum* compared with *L. tenue* populations. Boxplots show the distribution of *π* estimates in windows of 100 kb separately for each of eight *L. trigynum* populations (marked with a *L. tri.* prefix and a population number) and for each of eight *L. tenue* populations (marked with a *L. ten.* prefix and a population number, see supplementary table S1, Supplementary Material online). c) Comparison of genome-wide distribution of negative fitness effect estimates for 0-fold degenerate nonsynonymous mutations between *L. trigynum* (*n* = 8) and *L. tenue* (*n* = 8). Error bars represent the standard deviation derived from populations of each species. The fraction of mutations between species was significantly different for each category of *N*_e_s (0 > *N*_e_s > −1 = effectively neutral, −1 > *N*_e_s > −10 = moderately deleterious, −10 > *N*_e_s > −100 and *N*_e_s < −100 = strongly deleterious) (***P < 0.001, Wilcoxon rank-sum test). d) Comparison of *α* estimates for *L. tenue* and *L. trigynum* populations (***P < 0.001, Wilcoxon rank-sum test).

In addition to reduced herkogamy, *L. trigynum* has other traits typical of the floral selfing syndrome, including a smaller floral display (Fig. 1) and a 5-fold lower pollen:ovule ratio compared with *L. tenue* (Repplinger 2009), suggesting that self-pollination is its main mating strategy. If so, we would expect *L. trigynum* to be highly inbred compared with distylous SI *L. tenue*. To assess whether this was the case, we estimated the inbreeding coefficient using genome-wide polymorphism data from 224 individuals representing eight populations per species (average *n* = 14 individuals per population; supplementary table S1 and information, Supplementary Material online). We found that estimates of the inbreeding coefficient (*F*_IS_) for *L. trigynum* were significantly higher than those for *L. tenue* populations (Kruskal–Wallis test followed by Dunn's test with Benjamini–Hochberg adjustment of *P*-values, *P* < 0.05 for all except two *L. trigynum*—*L. tenue* population comparisons; summary of median *F*_IS_ values across populations: *L. tenue* mean = 0.09, SE = 0.03, *n* = 8, *L. trigynum* mean = 0.87, SE = 0.04, *n* = 8) (Fig. 4a). Assuming equilibrium (Wright 1969), the mean effective self-fertilization rate in *L. trigynum* is 0.93 (2 *F*_IS_/[1 + *F*_IS_]). These results show that *L. trigynum* is more inbred than *L. tenue*, consistent with expectations if SC and reduced herkogamy as a result of homostyly led to elevated selfing rates.

### Stronger Population Structure and Reduced Polymorphism in *L*. *trigynum* Compared with *L. tenue*

Transitions to self-fertilization are expected to result in reductions of the effective population size (*N*_e_), and thus reduced genetic diversity, as well as more pronounced population structure in selfers relative to outcrossers (Wright et al. 2013). To assess if the homostylous *L. trigynum* had reduced polymorphism levels genome-wide compared with the distylous *L. tenue*, we obtained windowed estimates of nucleotide diversity (*π*, 100 kb windows) for eight populations per species. In agreement with expectation, *L. trigynum* showed markedly lower genome-wide *π* than *L. tenue* (Fig. 4b; Kruskal–Wallis test followed by Dunn's test with Benjamini–Hochberg adjustment of *P*-values, *P* < 0.001 for all *L. trigynum*—*L. tenue* population comparisons; average *π-*values across populations: *L. tenue*: Mean = 1.3 × 10^−3^, SE = 1.4 × 10^−4^, *n* = 8, *L. trigynum*: mean = 5.3 × 10^−4^, SE = 1.2 × 10^−4^, *n* = 8).

**Fig. 5.**
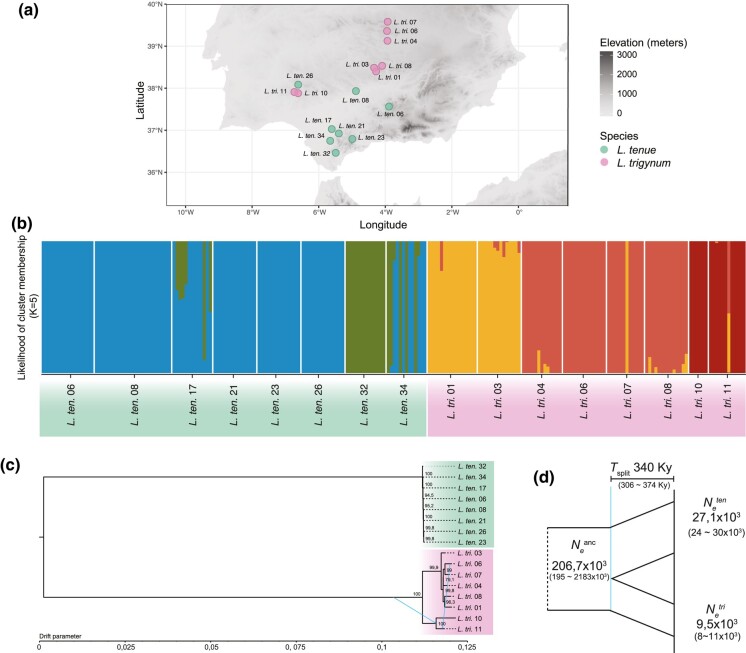
Population structure and demographic history of *L. trigynum* and *L. tenue*. a) Geographic origin of the Iberian populations of *L. trigynum* (*n* = 8; indicated by the prefix *L. tri.*) and *L. tenue* (*n* = 8; indicated by the prefix *L. ten.*) included in this study. b) Assignment of individuals to each of the five inferred ancestral clusters (*K* = 5) based on admixture models to describe population structure in ADMIXTURE. c) Maximum-likelihood tree inferred by TreeMix. Bootstrap values for each bifurcation and two migration edges within *L. trigynum* are shown. d) The demographic model inferred by *dadi*. Estimates of ancestral and current effective population sizes (in numbers of individuals) and the time of the split (in number of generations) are shown, with 95% confidence intervals based on 1,000 bootstrap replicates in parentheses.

Next, we investigated population structure based on our genome-wide polymorphism data (see Fig. 5a for geographical origin of the sampled individuals). Principal component analyses (PCA), population structure analyses (when *K* = 2), and TreeMix-based inference based on 76,934 Single Nucleotide Polymorphisms (SNPs) in noncoding regions (see Materials and Methods for details) showed that *L. tenue* and *L. trigynum* form clearly differentiated groups (Fig. 5c; supplementary fig. S6 and information, Supplementary Material online). Additionally, structure analyses with ADMIXTURE suggest that clustering is better defined in five groups (*K* = 5): three main regional populations in *L. trigynum* that largely coincide with their geographic origin, and two *L. tenue* populations (one of them widely distributed, and a second one present in the two southernmost populations; Fig. 5b and c).

We found the highest degree of genetic differentiation between *L. tenue* and *L. trigynum* populations (*F*_ST_ median = 0.96, 1st and 3rd quartile = 0.95 and 0.97), followed by *L. trigynum* (*F*_ST_ median = 0.37, 1st and 3rd quartile = 0.28 and 0.50), and finally *L. tenue* (*F*_ST_ median = 0.05, 1st and 3rd quartile = 0.03 and 0.05) (Kruskal–Wallis test followed by Dunn's test with Bonferroni corrected *P-*values, *P* < 0.001; supplementary fig. S7 and information, Supplementary Material online). TreeMix analyses (Fig. 5c) further suggested the absence of gene flow between *L. tenue* and *L. trigynum*, and stronger population structure within *L. trigynum* than in *L. tenue*. Overall, these results show low population structure within *L. tenue*, and more marked population structure within *L. trigynum* (Fig. 5b and c).

Finally, divergence population genomic analyses in *dadi* jointly estimating demographic parameters and inbreeding levels suggested that the split between *L. tenue* and *L. trigynum* occurred about 340 kya and was associated with a marked *N*_e_ reduction (Fig. 5d) and increased inbreeding in *L. trigynum* (*F*_IS_ = 0.88). Together, these results suggest that the transition to homostyly was associated with higher rates of self-fertilization and reduced *N*_e_, and occurred within a relatively recent evolutionary timeframe.

### Relaxed Purifying Selection Against Weakly Deleterious Mutations in *L*. *trigynum*

If the shift to selfing in *L. trigynum* led to relaxed purifying selection on nonsynonymous mutations, we might expect to observe an elevated ratio of nonsynonymous to synonymous polymorphism (*π*_N_*/π*_S_) in *L. trigynum* relative to *L. tenue*. In line with this expectation, we found that *π*_N_*/π*_S_ estimates were slightly higher in *L. trigynum* than in *L. tenue* (supplementary fig. S8 and information, Supplementary Material online; *L. tenue*: Mean = 0.40, SE = 0.02, *n* = 8, *L. trigynum*: Mean = 0.50, SE = 0.15, *n* = 8). To test if elevated *π*_N_/*π*_S_ in *L. trigynum* was due to weaker purifying selection, we estimated the distribution of negative fitness effects (DFE) of new nonsynonymous mutations in each population of *L. trigynum* and *L. tenue* using fastDFE v1.0.0 (Figure 4c). In line with the expectation that reduced *N*_e_ in selfers should lead to relaxed selection against weakly deleterious mutations (reviewed by Wright et al. 2013), we found that the proportion of new nonsynonymous mutations that are effectively neutral was significantly higher in *L. trigynum* (0 > *N*_e_s > −1: Median = 0.442) compared with *L. tenue* (0 > *N*_e_s > −1: Median = 0.24) (0 > *N*_e_s > −1: Wilcoxon rank-sum test, *P* < 0.001, *n* = 8 populations per species), suggesting that selection against weakly deleterious mutations is relaxed in *L. trigynum* relative to *L. tenue*. Moreover, we found a lower proportion of new nonsynonymous mutations with moderate and strongly deleterious effects in *L. trigynum* (−1 > *N*_e_s > −10: Median = 0.064, −10 > *N*_e_s > −100: Median = 0.074, *N*_e_s < −100: Median = 0.074) compared with *L. tenue* (−1 > *N*_e_s > −10: Median = 0.096, and −10 > *N*_e_s > −100: Median = 0.132, *N*_e_s < −100: Median = 0.524) (Wilcoxon rank-sum test, *P* < 0.001, *n* = 8 populations per species). Together, these results show that the efficacy of purifying selection on new nonsynonymous mutations is lower in *L. trigynum* than in *L. tenue*.

### Reduced Contribution of Positive Selection to Nonsynonymous Divergence in *L*. *trigynum*

We compared the proportion of nonsynonymous divergence driven by positive selection (*α*) in *L. tenue* and *L. trigynum* using a method that accounts for the impact of weakly deleterious mutations on *α* (Messer and Petrov 2013). We found that estimates of *α* were significantly higher for *L. tenue* than for *L. trigynum* (Wilcoxon rank-sum test, *n* = 8 populations per species, *P <* 0.001, median *α*: 0.40 vs. −0.18, for *L. tenue* and *L. trigynum*, respectively) (Fig. 4d). For all except one *L. tenue* population, 95% confidence intervals of *α* estimates did not overlap with zero (supplementary fig. S9 and information, Supplementary Material online), indicating that there is evidence for adaptive nonsynonymous divergence in this species. In contrast, for *L. trigynum*, all 95% confidence intervals encompassed zero, suggesting that there is no evidence for significant adaptive nonsynonymous divergence in *L. trigynum* (supplementary fig. S9 and information, Supplementary Material online).

## Discussion

The genetic basis and evolutionary significance of distyly have interested many generations of biologists (e.g. Darwin 1876, 1877; Bateson and Gregory 1905; Ernst 1936). The study of distyly and its breakdown has further inspired development of theory (e.g. Dowrick 1956; Charlesworth and Charlesworth 1979a, b; Lloyd and Webb 1992) and vast amounts of empirical work (reviewed by Ganders 1979; Barrett 1992; Barrett 2019). In recent years, new genomic tools have enabled the generation of high-quality genome assemblies of nonmodel plant species. This development has opened up the possibility to use genomic approaches to test long-standing hypotheses on the evolution and loss of distyly in a range of systems where such work was not possible before.

Although *Linum* is a classic system for the study of distyly (Darwin 1863), few studies have investigated the genetic causes and population genomic consequences of loss of distyly and evolution of homostyly in *Linum*. Here, we generated a high-quality reference genome assembly of the homostylous species *L. trigynum* and used it to test hypotheses on the transition to homostyly, and to investigate the population genomic effects of this shift in terms of inbreeding levels, patterns of polymorphism, and the efficacy of natural selection genome-wide.

While earlier theory posited that rare recombination events between the recessive and dominant alleles at the *S*-locus were the cause for distyly breakdown (Dowrick 1956; Charlesworth and Charlesworth 1979a), recent findings have indicated that distyly *S-*loci harbor presence–absence variation and that thrums are predominantly hemizygous at the *S*-locus rather than heterozygous (Li et al. 2016; Shore et al. 2019; Gutiérrez-Valencia et al. 2022a; Fawcett et al. 2023; Yang et al. 2023; Zhao et al. 2023). These findings make previous models of breakdown less likely and indicate that mutations either at the *S-*locus or at unlinked modifier loci are more plausible causes for the evolutionary shift from distyly to homostyly.

The presence of a genomic region in *L. trigynum* homologous to the thrum-specific dominant *S-*haplotype of *L. tenue* strongly suggests that breakdown of distyly first occurred in a thrum individual. This finding allowed us to identify candidate sequence and gene expression changes that might have contributed to the evolution of homostyly in *L. trigynum*. Although we found a premature stop codon in *LtTSS1*, which is a strong candidate gene for style length and possibly female SI in *Linum* (Gutiérrez-Valencia et al. 2022a), this mutation was not fixed in *L. trigynum*, suggesting that loss of distyly has different genetic basis in this species. The fact that *LtTSS1* alleles with and without this mutation segregate in homostylous populations suggests that it might not have altered stylar condition, possibly because it is located near the end of the coding sequence and does not disrupt the two conserved motifs in the protein encoded by this gene (Ushijima et al. 2012).

Our differential expression analyses showed that *LtWDR-44*, a candidate gene for anther position and/or pollen functioning, was the only *S*-linked gene downregulated in floral buds of *L. trigynum* compared with *L. tenue*. Pollination assays further showed that *L. trigynum* pollen tubes can grow in styles of *L. tenue* thrums but not in those of pins, consistent with expectations if downregulation of *LtWDR-44* in *L. trigynum* resulted in a switch in the male SI reaction from thrum-type to pin-type, a change that is likely to have rendered *L. trigynum* SC. These results suggest that mutations resulting in downregulation of the expression of *LtWDR-44* are a plausible cause of the evolution of SC and homostyly in *L. trigynum* via altered pollen SI and possibly effects on anther height, and should be subject to further functional study. Differences in TE content of the promoter region of *LtWDR-44* and especially the presence of LTR–Ty3 retrotransposons only in *L. trigynum* and not in *L. tenue* are of special interest in this respect, given that insertions of LTR–Ty3 elements in promoter regions have been associated with reduced gene expression (e.g. Steige et al. 2015; Castanera et al. 2023). Moreover, previous work showed that loss of homomorphic SI (e.g. in *Arabidopsis thaliana*) might have evolved in association to mutations at the 5′ flanking region of the male SI component of the *S*-locus (i.e. *SCR*) by reducing its expression in anthers (e.g. Tsuchimatsu et al. 2017). It remains to be studied if this path to SC is feasible and frequent in species with heteromorphic SI systems.

Future work could expand our understanding of the breakdown of distyly in *L. trigynum* by investigating whether the alteration of *LtWDR-44* might simultaneously alter male SI reaction and contribute to decreased herkogamy, or if more complex evolutionary pathways could have led to the evolution of homostylous flowers. Studies in *Turnera* support the plausibility of this final scenario by showing that the *S*-linked *YUCG* gene determines the functioning and size of pollen, but does not affect stamen length (Henning et al. 2022). Our finding of downregulation of *LtWDR-44* associated with SC and a switch from thrum- to pin-type male SI in *L. trigynum* constitutes an interesting contrast to the mode of loss of distyly in *Primula* and *Fagopyrum*, which involved mutations at *S-*linked candidate genes for style length and female SI function (Huu et al. 2016, 2022; Mora-Carrera et al. 2023; Fawcett et al. 2023).

Theory predicts that SC long homostyles should be favored during establishment of homostyly (Dowrick 1956; Charlesworth and Charlesworth 1979a), as they can spread faster than short homostyles. Work in *Primula* (Huu et al. 2016, 2022; Mora-Carrera et al. 2023) and *Fagopyrum* (Fawcett et al. 2023) has shown that shifts to homostyly in both lineages were most likely precipitated by mutations at *S*-linked genes that simultaneously govern pistil elongation and female SI. These results are in line with theoretical predictions (Dowrick 1956; Charlesworth and Charlesworth 1979a). In contrast, our findings in *L. trigynum* so far do not conform to these theoretical expectations, but rather suggest that mutations leading to a SC short homostyle have been fixed in this species. There are several possible reasons, which are not mutually exclusive, for why this might be the case. First, in the absence of long homostyles, short homostyles would still have an advantage over distylous individuals under conditions that favor selfing (Dowrick 1956; Charlesworth and Charlesworth 1979a), including the establishment of new populations in localities where pollination opportunities are scarce (Baker 1955; Stebbins 1957). In *Linum,* homostyly is associated with range expansion outside of the ancestral area in the Western Palearctic (Maguilla et al. 2021), suggesting that such scenarios might be plausible, including for *L. trigynum* which has a much wider geographical range than distylous *L. tenue*. If mutations resulting in homostyly are rare, as we might expect given the relatively low levels of genome-wide polymorphism in *L. trigynum* and *L. tenue*, then chance events might determine whether short or long homostyles are first formed and exposed to selection.

Second, mutational biases (reviewed in Shimizu and Tsuchimatsu 2015) might affect the rate of appearance of each of the two types of homostyles. For instance, we might expect SC long homostyles to evolve more readily if the mutation rate at genes affecting style length/female SI is higher than that at genes affecting anther height/male SI. Conversely, if the mutation rate is higher at genes affecting anther height/male SI, we could expect SC short homostyles to evolve more readily. While the length of regulatory regions affecting style length/female SI or anther height/male SI is generally not known in any system and should ideally also be considered, it is interesting to note that in *P. vulgaris*, the gene for female SI and style length is substantially longer than the gene affecting anther position (style length/female SI gene *CYP^T^* aka *CYP734A50:* 68 kb vs. anther height gene *GLO^T^*, aka *GLO2*: 25 kb; Li et al. 2016), whereas in *L. tenue* the situation is the opposite (*LtTSS1* gene length ∼1.9 kb, *LtWDR-44* gene length ∼11.0 kb). Thus, if mutational biases resulting from mutational target size alone are driving differences among systems in the type of homostyly that evolves, we might expect style length/female SI genes to be more frequently involved in *Primula*, and less frequently in *Linum*. To test this hypothesis, it would be beneficial to take advantage of the multiple independent transitions from distyly to homostyly that have been documented in *Linum* (at least six transitions inferred in Ruiz-Martín et al. 2018; note that in *Linum* it is not generally known a priori whether homostyles are short or long homostyles). In the case of *L. trigynum*, where mutations at *S*-linked coding sequences are likely not associated to distyly breakdown, differences in the length of *cis*-regulatory regions of *S*-linked genes could have similarly determined which functions of distyly were more likely to be affected via altered expression. It remains to be determined if the regulatory sequences of *LtWDR-44* are larger than those of *LtTSS1*, and if this feature could have increased the chances of regulatory mutations affecting *LtWDR-44*.

Third, it is possible that the function of *S-*locus genes in *Linum* render a transition to homostyly via long homostyles more difficult than in *Primula*. In the absence of functional genetic studies of *LtTSS1,* it remains uncertain whether loss-of-function mutations at this gene could simultaneously alter both style length and female SI, as is the case in *Primula*. A caveat regarding the potential causes of loss of distyly in *L. trigynum* that we have identified is that we cannot completely rule out a contribution of mutations at non-*S*-linked loci to reduced herkogamy and SC in *L. trigynum* (Mather and De Winton 1941; Ganders 1979). Unfortunately, such studies are precluded here by the marked genome-wide differentiation between *L. trigynum* and *L. tenue* which prevents identification of genetic variants associated with floral morph using association mapping. Additionally, we were unable to obtain viable offspring from crosses of *L. trigynum* and *L. tenue*, preventing genetic mapping in interspecific F2 mapping populations. Functional assays should instead be used to validate candidate genetic changes that triggered loss of distyly in *L. trigynum*.

Transitions from distyly to homostyly are expected to frequently result in elevated self-fertilization rates, with major consequences for genetic variation and the efficacy of selection. To elucidate the timing of the split between *L. tenue* and *L. trigynum*, and the genomic consequences of evolution of homostyly, we analyzed 224 whole-genome sequences of individuals from eight populations each of *L. trigynum* and *L. tenue*. We found that homostyly was associated with predominant self-fertilization, and that the split between *L. trigynum* and *L. tenue* happened *ca.* 340 kya (∼12.5 *N*_e_ generations ago, in terms of *L. tenue N*_e_), setting an upper bound for the timing of evolution of homostyly. This estimate broadly coincides with the occurrence of major transformations in European flora attributed to the dramatic climatic oscillations that took place during the Quaternary, which resulted in range shifts for multiple plant lineages (Davis and Shaw 2001). Importantly, transitions to self-pollination have been acknowledged as adaptations that allow trailing edge populations to expand their range, especially if pollinator service is unreliable (Levin 2012), as has been inferred for other regions impacted by climate dynamics of the Quaternary (e.g. Groom et al. 2014). Indeed, *Usia* bee flies, which are relevant pollinators of various *Linum* species in the Mediterranean basin (Johnson and Dafni 1998; Ruiz-Martín et al. 2018; Pérez-Barrales and Armbruster 2023), significantly reduce their foraging capacity outside of their thermal tolerance range (21 to 31 °C) (Orueta 2002). Therefore, it is likely that homostyly in *L. trigynum* was advantageous in the context of range expansion, past climatic instability and unreliable pollination services. Evidence for an association between range expansion and homostyly has been found in a recent phylogenetic study of the diversification of *Linum* (Maguilla et al. 2021).

Our population genomic analyses show that, in line with some of the most frequently reported consequences of shifts to selfing (reviewed in Cutter 2019), *L. trigynum* is more inbred, less genetically diverse, and shows stronger population structure than *L. tenue*. Reductions in *N*_e_ resulting from bottlenecks and an increased impact of linked selection due to elevated inbreeding are expected to result in reduced efficacy of selection against weakly deleterious mutations in selfers (Wright et al. 2013; Burgarella and Glémin 2017; Cutter 2019). In line with this prediction, we found evidence for weaker purifying selection in *L. trigynum* than in *L. tenue* based on analyses of the distribution of fitness effects of new nonsynonymous (0-fold degenerate) mutations.

Selfing has been proposed to reduce the potential for adaptation (Stebbins 1957), and empirical population genomic studies have found evidence for slower rates of adaptive evolution in derived selfing lineages (e.g. Slotte et al. 2010; Burgarella et al. 2015). Using a version of the McDonald–Kreitman test that corrects for biases resulting from weakly deleterious mutations (Messer and Petrov 2013), we found that the proportion of nonsynonymous fixations driven by positive selection was significantly lower (and not significantly different from zero) for the homostylous *L. trigynum*, in contrast to the distylous *L. tenue* which had evidence for a significant contribution of positive selection to nonsynonymous divergence. While detailed analyses separately assessing selection on specific classes of genes, such as those involved in the evolution of the selfing syndrome or pollen competition (Gutiérrez-Valencia et al. 2022b) would be warranted for a more complete understanding of the impact of shifts to selfing on selection across the genome, our results so far demonstrate genomic signatures consistent with relaxed purifying and positive selection on nonsynonymous mutations in association with loss of distyly. These results are in line with those in a previous study on the genomic impact of loss of distyly in *Primula* (Wang et al. 2021), and with a wealth of studies on the genomic impact of shifts to selfing in other plant lineages (e.g. Slotte et al. 2010, 2013; Laenen et al. 2018; Mattila et al. 2020; Yi et al. 2022). Comparative population genomic analyses of multiple shifts from distyly to homostyly in *Linum* will be valuable to better understand the general population genomic effects of this major evolutionary transition.

Our study represents the most comprehensive assessment of the genetic basis and population genomic consequences of the shift from distyly to homostyly in *Linum* so far. By generating a chromosome-scale genome assembly, we have enabled tests of long-standing hypotheses on the evolution of homostyly using genomic methods. We show that the genetic basis of homostyly is likely to be different in *L. trigynum* than in *Primula* and *Fagopyrum* and discuss potential reasons for such differences. Our results demonstrate pervasive genome-wide consequences of the shift to homostyly and elevated selfing rates, including reduced genome-wide polymorphism, stronger population structure, and a reduced efficacy of both purifying and positive selection. Taken together, this study expands the study of shifts from distyly to homostyly to a new system, provides a basis for future research on the pathways associated with loss of distyly, and broadens our knowledge on the evolutionary genomic consequences of shifts to homostyly and self-fertilization.

## Materials and Methods

### Plant Material and Sequencing

For genome sequencing and population genomic analyses, seeds and leaves of *L. trigynum* and *L. tenue* were sampled in 16 different localities in southern Spain, representing eight populations per species (supplementary table S1 and information, Supplementary Material online). Plants were grown in a controlled climate chamber at Stockholm University (Stockholm, Sweden) set to 16 h light at 20 °C:8 h dark at 18 °C, 60% maximum humidity, 122 μE light intensity. We extracted DNA from 224 individuals for whole-genome short-read sequencing on an Illumina NovaSeq 6000 on a S4 flowcell (150-bp paired-end reads, v1.5 sequencing chemistry). For long-read and linked-read sequencing we extracted high molecular weight (HMW) DNA from young leaves using the CTAB method (as in Fulton et al. 1995), followed by two purification steps with Genomic tip 500/G (QIAGEN, Germany). To generate a high-quality genome assembly, HMW DNA of individual *Ltri*. 6-30-2 was sequenced on 2 Sequel II SMRT cells in HiFi mode, which led to the production of 27 Gb of HiFi data. This was supplemented with Dovetail Hi-C sequencing data (OmniC) for the same individual. For linked-read sequencing of two samples of *L. trigynum* (*Ltri.* 1-1-1 and *Ltri*. 1-42-2), four samples of *L. tenue* (*Lten*. CL-3-1, *Lten*. CL-75-1, *Lten*. STM-5-2, and *Lten.* STM-30-1), and one sample of the outgroup *L. maritimum* (*Lmar.* 08 thrum) we used the Chromium Genome Library preparation kit with sequencing on an Illumina HiSeqX system (paired-end 150 bp read length, v2.5 sequencing chemistry). For genome annotation, we extracted total RNA from stems, leaves, floral buds, and mature flowers of individual *Ltri*. 6-30-2, and for differential expression analyses, we extracted total RNA from floral buds and leaves of *L. tenue* and *L. trigynum* (Sample size: *L. tenue* thrum = 6 individuals, pin = 4 individuals, *L. trigynum* homostyle = 5 individuals) using the RNeasy Plant Mini Kit (QIAGEN, Germany). Libraries were sequenced on an Illumina NovaSeq S1 Sequencing System to produce paired-end 150 bp read length reads. Full details on plant growth, sampling, extraction and sequencing are given in supplementary methods, Supplementary Material online.

### De Novo Genome Assemblies

HiFi PacBio subreads were assembled with IPA (v1.3.2) (https://github.com/PacificBiosciences/pbipa) using reads with QV20 or higher, which led to a preliminary assembly of 103 primary contigs (total length = 498.60 Mb) and 2,280 associated contigs (total length = 100.57 Mb). To check for primary contigs that should be identified as haplotigs we used Purge Haplotigs (Roach et al. 2018). Illumina short reads obtained from individual *Ltri*. 6-30-2 were mapped to the primary IPA assembly using minimap2 (Li 2018), and the alignments were processed with the function purge_haplotigs to generate a coverage histogram for contigs. The resulting histogram showed a unimodal distribution, suggesting that purging was not required. The assembly was scaffolded using Dovetail Hi-C data using 3D-DNA scaffolding (Dudchenko et al. 2017), which was prefiltered to only keep contacts with a mapping quality higher than 30. The expected haploid number of chromosomes in *L. trigynum* is 10 (Pastor et al. 1990), and the resulting scaffolded assembly consisted of 13 pseudochromosomes (N50 = 47.03 Mb, Length = 498.10 Mb). This assembly was further edited to correct three misassemblies that were not supported by mapping of our PacBio reads to the *L. trigynum* assembly. The edited *L. trigynum* genome assembly was polished two times with Pilon (Walker et al. 2014) using Illumina short reads of the same individual. We screened the genome assembly for regions with high coverage and with similarity with the NCBI Plastid database (https://www.ncbi.nlm.nih.gov/genome/organelle/). We detected and hard-masked one region with plastid contamination on chromosome 6 (positions 59,960,001 to 60,370,000). We visualized broad-scale genome synteny between our *L. trigynum* and *L. tenue* (Gutiérrez-Valencia et al. 2022a) assemblies by aligning genome assemblies using minimap2 v2.4 (Li 2018) and plotting alignments larger than 100 kb using the R package circlize (Gu et al. 2014) (Fig. 2a).

Genomic linked-read sequences (10× Genomics) of two *L. trigynum* individuals, four *L. tenue* individuals and one *L. maritimum* individual were assembled with the Supernova pipeline (https://github.com/10XGenomics/supernova) using default parameters to obtain the output type pseudohap2. Furthermore, we included three additional *L. tenue* linked-read assemblies from Gutiérrez-Valencia et al. (2022a).

### Genome Annotation

The annotation of *L. trigynum* was made with TSEBRA (Gabriel et al. 2021) which combines the gene prediction of BRAKER1 (Hoff et al. 2016) and BRAKER2 (Brůna et al. 2021). BRAKER1 predicts genes using RNA-seq data; therefore, we trimmed the raw reads from four different tissues (leaves, stems, floral buds, and mature flowers) with fastp (Chen et al. 2018), after we aligned them using STAR (Dobin and Gingeras 2015). BRAKER2 predicts genes using protein databases; therefore, we used protein data from *L. tenue* (Gutiérrez-Valencia et al. 2022a) and four additional Malpighiales species (*Linum usitatissimum*, *Manihot esculenta*, *Populus trichocarpa*, and *Salix purpurea*) downloaded from phytozome (https://phytozome-next.jgi.doe.gov/) (Goodstein et al. 2012). We used AGAT (https://github.com/NBISweden/AGAT) to extract the coding DNA sequences needed to assess the genome completeness with BUSCO v5 (Manni et al. 2021). Repetitive elements were identified using RepeatMasker (Smit et al. 2013) using a custom repeat library modeled using RepeatModeler (Smit and Hubleyo 2008).

We carefully reannotated the *S-*locus region in *L. tenue* and the corresponding region in *L. trigynum* to improve on the automated annotation and fully consider RNA-Seq evidence. This was necessary as the original annotation of genes at the *S-*locus in *L. tenue* was based on Augustus (Stanke et al. 2008) predictions without including RNA-Seq evidence. Here, we therefore reannotated the *L. tenue S-*locus using RNA-Seq data from the genome sequenced thrum individual generated in (Gutiérrez-Valencia et al. 2022a) and we likewise used *L. trigynum* RNA-Seq evidence to annotate the corresponding region in *L. trigynum*. For this purpose, we first identified transcripts with StringTie v2.1.4 (Pertea et al. 2015) using the RNA-Seq data from (Gutiérrez-Valencia et al. 2022a). Second, we predicted proteins using TransDecoder v5.7.0 (https://github.com/TransDecoder/). Third, we visually inspected the annotated genes using IGV v2.12.3 (Thorvaldsdóttir et al. 2013) retaining those well-supported by RNA-Seq evidence.

In comparison with the previous *L. tenue* annotation (Gutiérrez-Valencia et al. 2022a), two genes (*indelg1* and *LtTSS1*) were modified, leaving a total of nine genes at the dominant allele of the *L. tenue S-*locus. Six genes with high similarity to repeats (*indelg3*, *indelg5*, *indelg6*, *indelg7*, *indelg8*, and *indelg9*) that were not analyzed in Gutiérrez-Valencia et al. (2022a) were not retained in our updated annotation. In particular, our new annotation supported a one-exon gene structure of *LtTSS1* which was validated using PCR-based assays on cDNA and genomic DNA (supplementary methods, fig. S3, and information, Supplementary Material online).

In the *L. trigynum S-*locus region, we removed 22 genes annotated by TSEBRA because they were not well-supported by RNA-Seq evidence and we manually reannotated two genes (*indelg2* and *LtTSS1*) in order to have the same intron–exon structure of the orthologous genes in *L. tenue*. Finally, we used NUCmer (Kurtz et al. 2004) to identify orthologous regions between *L. tenue* and *L. trigynum* in the *S-*locus region (Fig. 2d).

### Identification of Candidate Loss of Function S-Linked Mutations

Using minimap2 (Li 2018), we mapped the sequence of the *L. tenue* distyly *S*-locus (Gutiérrez-Valencia et al. 2022a) against the genome assembly of *L. trigynum* to identify and extract the sequence homologous to this region (Fig. 2b). The same approach was used to retrieve contigs containing the *S-*locus from 10× genomics supernova assemblies (two *L. trigynum* and six *L. tenue*). We conducted BLAST analyses (Camacho et al. 2009) to identify the sequences of *S*-locus genes in each assembly. Sequences of each gene were independently aligned using MUSCLE v3.8.31 (Edgar 2004), and only coding sequences were kept for further analyses. Coding sequences were aligned using codon-aware alignment in webPRANK (Löytynoja and Goldman 2010). We inspected each alignment using AliView (Larsson 2014) to identify major-effect mutations (nonconsensus splice sites and premature stop codons). Estimates of mean synonymous (*d_S_*) and nonsynonymous divergence (*d_N_*) between *L. tenue* and *L. trigynum* were obtained in MEGA X (Kumar et al. 2018; Stecher et al. 2020) using the Nei–Gojobori model (Nei and Gojobori 1986), with standard error estimates obtained using 1,000 bootstrap replicates. Ambiguous codon positions were removed for each sequence pair.

### Sequence Processing, Mapping, Variant Calling, and Filtering

Illumina short reads from 224 individuals representing populations of *L. tenue* and *L. trigynum* were quality and adaptor trimmed with *bbduk* from BBMap/BBTools (Bushnell 2015). Trimmed paired-end reads were mapped to the *L. tenue* genome assembly using BWA-MEM (v0.7.17) (Li 2013). Alignments of *L. tenue* and *L. trigynum* short reads to the *L. trigynum* reference genome were used for coverage analyses focusing on the *S-*locus region. For joint inference of demographic history and analyses of population structure, short-read sequences of both species were mapped to the *L. tenue* genome assembly (Gutiérrez-Valencia et al. 2022a). For all remaining analyses, sequences of each species were mapped to their corresponding reference genome, and processed independently in downstream analyses leading to variant calling.

Alignments with mapping quality lower than 20 were discarded, and we used *MarkDuplicates* from Picard tools v2.0.1 (Broad Institute 2019) to remove duplicated reads from the alignment. The resulting alignments were used to obtain genotype likelihoods with *mpileup*, and variants (SNPs/INDELs) and invariant sites were identified by samtools/bcftools, using the model for multiallelic and rare-variant calling (Danecek and McCarthy 2017). The variant call format (VCF) file was processed to keep only biallelic SNPs and invariant sites, and then filtered based on the maximum proportion of missing data (pm = 0.1) and read depth (5 < dp < 200). To avoid false heterozygous calls based on a low number of alternate alleles, we used a combination of allele balance and coverage filtering, which has previously been successful for highly repetitive plant genomes (see Laenen et al. 2018, Gutiérrez-Valencia et al. 2022b for a detailed description).

### Coverage Analyses

To investigate differences in depth of coverage between pin (*n* = 25), thrum (*n* = 26) (*L. tenue*), and homostyle (*n* = 104) (*L. trigynum*) at the *S*-locus, sequences mapped to the *L. trigynum* genome assembly were processed to remove repetitive regions identified with RepeatMasker (Smit et al. 2013) using the *L. usitatissimum* repeat library. We used BEDTools (Quinlan and Hall 2010) to estimate coverage for 50 kb windows across the genome. Estimates were further processed in R to estimate normalized mean coverage across windows, and differences between morphs were tested using a Kruskal–Wallis test, followed by a post hoc Dunn's test with Bonferroni correction for multiple testing.

### Haplotype Network Analyses

To investigate whether loss of distyly in *L. trigynum* might have occurred repeatedly, we conducted a haplotype network analysis of two genes at the *S-*locus, *LtTSS1* and *LtWDR-44*. We used the genome annotations of *L. tenue* and *L. trigynum* species to extract coding sequences for these two genes. We then extracted the corresponding sequences from our short-read data using bam2consensus, a tool from the package tool bambam v.1.4 (Page et al. 2014). In total, 67 *L. tenue* and 100 *L. trigynum* individuals had sufficient coverage and were included in this analysis. We aligned all sequences using codon-aware alignment in PRANK (Löytynoja and Goldman 2010). Haplotypes and haplotype network were assessed using the R package pegas v.1.2 (Paradis 2010).

### Differential Expression Analyses

RNA-Seq raw reads from floral buds and leaves were processed with the function bbduk from BBMap/BBTools (Bushnell 2015) for quality and adapter trimming (parameters *k* = 2, mink = 11, ktrim = r, minlength = 50, qtrim = rl, trimq = 20, hdist = 1, tbo, tpe). Reads were mapped and quantified with STAR (Dobin and Gingeras 2015), using the genome reference and the longest isoform per transcript from our updated annotation of *L. tenue*. Multimapping reads were discarded by using the flag outFilterMultimapNmax = 1. Files listing counts mapped to each feature (ReadsPerGene.out.tab) were further processed in R to conduct differential expression analyses using the package DESeq2 (Love et al. 2014). Two independent contrasts were conducted for floral buds and leaves separately: Homostyles were first contrasted to thrums, and then to pins. We corrected for multiple testing with the Benjamini–Hochberg method, and genes with an adjusted Log_2_-fold change >|1.5| and *P* < 0.01 were considered significantly differentially expressed.

### Pollination Assays

To test the functionality of male and female SI in *L. trigynum*, we conducted controlled reciprocal crosses of *L. trigynum* (*n* = 2 individuals) to both pin (*n* = 2 to 5) and thrum (*n* = 2 to 4) morphs of *L. tenue.* For comparison, we also conducted self-pollination of *L. trigynum*, compatible (pin × thrum, thrum × pin) and incompatible pollinations (thrum × thrum, pin × pin) in *L. tenue* and negative controls without pollination. We did three technical replicates of each type of cross of two individuals. For pollination, whole pistils were removed from mature flower buds or recently opened flowers, placed on agar plates and hand-pollinated. Pollen tube growth was observed after 4 h. For pollen tube staining, we adapted a protocol by Mori et al. (2006). Specifically, hand-pollinated pistils were fixed with 9:1 ethanol: acetic acid solution 4 h after pollination. Pistils were then hydrated with an ethanol series (70%, 50%, and 30% ethanol) and softened overnight in 1 M NaOH. Pollen tube growth was observed under an UV fluorescence microscope (Olympus BX60) after staining with 0.1% (*w*/*v*) aniline blue solution in 100 mM K_3_PO_4_ and 2% glycerol.

### Population Structure and Timing of Split

The filtered VCF was pruned based on linkage disequilibrium (*r*^2^) in PLINK (Chang et al. 2015) (parameters: window size in kb = 50, variant count to shift the window at the end of each step = 5, pairwise *r*^2^ threshold = 0.5) prior to conducting structure and demographic analyses. We used the function—*pca* also implemented in PLINK (Chang et al. 2015) to conduct a PCA on SNPs in the pruned VCF. The same data set was used for the structure analysis in ADMIXTURE (Alexander et al. 2009) using values of *K* ranging from 2 to 16. The most likely number of subpopulations in the population was determined after identifying the *K* value with the lowest cross-validation error. The results of both the PCA and structure analyses were plotted in R (R Core Team 2021). Weighted *F*_ST_ values were calculated using the Weir and Cockerham estimator (Weir and Cockerham 1984) implemented in VCFTools using the function—*weir-fst-pop* (Danecek et al. 2011). Pairwise *F*_ST_ values were then compared within and between species using a Kruskal–Wallis test followed by Dunn's test, and *P*-values were corrected using the Bonferroni method in R (R Core Team 2021). We investigated historical relationships among populations using TreeMix (Pickrell and Pritchard 2012) with 0 to 5 migration edges, running 100 iterations to get the optimal number of migration edges. Using the evanno method implemented in the R package OptM (Evanno et al. 2005; Fitak 2021), we got an optimum of two migration edges, which were between populations of *L. trigynum*. We further ran 60 iterations with two migration edges to ensure we had the maximum-likelihood tree, as well as 1,000 bootstrap replicates to obtain confidence intervals. No admixture was detected between the species. Finally, we used an extension of *dadi* (Gutenkunst et al. 2009) by Blischak et al. (2020) to coestimate inbreeding and demographic parameters of a simple split model for *L. tenue* and *L. trigynum*. For simplicity, we only included one population each of *L. tenue* and *L. trigynum* (populations 32 and 11, respectively) in this analysis. We obtained 95% confidence intervals of parameter estimates in *dadi* using the Godambe Information Matrix to account for linkage, with 1,000 bootstrap replicates and the eps step size parameter set to 1 × 10^−5^. We assumed a mutation rate of 7 × 10^−9^ (Ossowski et al. 2010) and a generation time of 1 year per generation when converting demographic parameters to units of number of individuals and years.

### Estimates of Inbreeding and Polymorphism

We estimated the inbreeding coefficient (*F*_IS_) (Wright 1949) to assist our understanding of the prevalence of selfing in *L. trigynum* using the option—*het* in VCFTools (Danecek et al. 2011). Nucleotide polymorphism (*π*) was estimated in 100 kb windows per population using pixy (Korunes and Samuk 2021). Statistical testing for significant differences in *F*_IS_ and windowed *π* across populations was conducted in R (R Core Team 2021).

### Estimates of Purifying and Positive Selection

We investigated purifying selection at coding sequences using the annotation of both *L. tenue* (Gutiérrez-Valencia et al. 2022a) and *L. trigynum*. We calculated *π_N_*/*π_S_* using pixy with the options *−bed_file* and *−sites_file* to estimate *π* on a per-gene basis and by restricting the analyses to 0-fold and 4-fold sites. *π_N_* and *π_S_* were computed in R to obtain and compare values of *π_N_*/*π_S_*. These sites were identified using the python script NewAnnotateRef.py (https://github.com/fabbyrob/science/tree/master/pileup_analyzers; Williamson et al. 2014) ran separately on our annotated high-quality long-read *L. tenue* and *L. trigynum* assemblies, considering only the longest transcript per gene. Finally, estimates of DFE for each population were obtained using fastDFE (v1.0.0) (https://github.com/Sendrowski/fastDFE; Sendrowski and Bataillon 2024), a python implementation of polyDFE (Tataru et al. 2017) which supports deleterious DFE inference from folded frequency spectra. We used the model “GammaExpParametrization” which models the DFE under a Γ distribution. In the model, we parametrized the folded SFS using the nuisance parameters with the option “get_demography” to account for demographic history effects. We ran 200 iterations to get the highest maximum-likelihood fit, while confidence intervals were obtained with 300 bootstrap replicates. As before, 0-fold degenerate sites were considered to be under stronger purifying selection than 4-fold degenerate sites which were assumed to evolve neutrally. Site frequency spectra for 0- and 4-fold sites were obtained with easySFS (https://github.com/isaacovercast/easySFS) which uses a modified implementation of *dadi* (Gutenkunst et al. 2009) with the option “−a” to keep all SNPs. DFE results were summarized in four bins representing the proportion of new mutations evolving as effectively neutral (0 > *N*_e_s > −1), moderately (−1 > *N*_e_s > −10), and strongly deleterious (−10 > *N*_e_s > −100) and (−100 > *N*_e_s). As we were particularly interested in the proportion of effectively neutral mutations (0 > *N*_e_s > −1), which are not well represented in the output of the default binning script provided with fastDFE, which instead plots (0 > *4N*_e_s > −1), we rescaled the bin limits to achieve a more biologically interpretable representation of the DFE. The proportion of mutations in each category was compared between species using Wilcoxon rank-sum tests in R (R Core Team 2021).

To compare the contribution of positive selection to nonsynonymous divergence in *L. tenue* and *L. trigynum*, we estimated the proportion of adaptive nonsynonymous divergence (*α*) using a method that accounts for and corrects for the impact of weakly deleterious nonsynonymous polymorphism (Messer and Petrov 2013). To obtain divergence differences, we first conducted a pairwise alignment of the reference genomes of *L. tenue* and *L. trigynum*, and then aligned them to a draft linked-read assembly of the distylous outgroup species *L. maritimum* (supplementary table S3 and information, Supplementary Material online), using AnchorWave v. 1.1.1 (Song et al. 2022). We inferred ancestral states at variable sites in each focal species (*L. trigynum* or *L. tenue*) using a maximum-likelihood-based method (Keightley and Jackson 2018). Finally, we counted 4-fold and 0-fold sites, divergence differences and polymorphisms using fastDFEv1.0.0. Based on these counts, we conducted the McDonald and Kreitman test in iMKT (Murga-Moreno et al. 2019). We used Messer and Petrov's (2013) asymptotic method to obtain point estimates and 95% confidence intervals of *α* for each *L. tenue* and *L. trigynum* population. We conducted a Wilcoxon rank-sum test in R (R Core Team 2021) to compare *α* estimates across *L. tenue* and *L. trigynum*.

## Supplementary Material

msae087_Supplementary_Data

## Data Availability

All sequencing data, genome assemblies, and their annotation produced in this study have been uploaded to the European Nucleotide Archive (ENA) (https://www.ebi.ac.uk/ena/) under study accession number PRJEB67577. Annotation files are additionally available at the SciLifeLab Data Repository (doi:10.17044/scilifelab.24340378; doi:10.17044/scilifelab.24343000; doi:10.17044/scilifelab.24324310; and doi:10.17044/scilifelab.24324382).

## References

[msae087-B1] Alexander DH, Novembre J, Lange K. Fast model-based estimation of ancestry in unrelated individuals. Genome Res. 2009:19(9):1655–1664. 10.1101/gr.094052.109. 19648217 PMC2752134

[msae087-B2] Armbruster WS, Pérez-Barrales R, Arroyo J, Edwards ME, Vargas P. Three-dimensional reciprocity of floral morphs in wild flax (*Linum suffruticosum*): a new twist on heterostyly. New Phyt. 2006:171(3):581–590. 10.1111/j.1469-8137.2006.01749.x. 16866960

[msae087-B3] Ashworth L, Aguilar R, Martén-Rodríguez S, Lopezaraiza-Mikel M, Ávila-Sákar G, Rosas-Guerrero V, Quesada M. Pollination syndromes: a global pattern of convergent evolution driven by the most effective pollinator. In: Pontarotti P, editor. Evolutionary biology: biodiversification from genotype to phenotype. Cham: Springer; 2015. p. 203–224.

[msae087-B4] Baker HG . Self-compatibility and establishment after “long-distance” dispersal. Evolution. 1955:9:347–349.

[msae087-B5] Barrett SCH . Evolution and function of heterostyly. Berlin: Springer-Verlag; 1992.

[msae087-B6] Barrett SCH . Understanding plant reproductive diversity. Philos T R Soc B. 2010:365(1537):99–109. 10.1098/rstb.2009.0199. PMC2842705 20008389

[msae087-B7] Barrett SCH . ‘A most complex marriage arrangement’: recent advances on heterostyly and unresolved questions. New Phyt. 2019:224(3):1051–1067. 10.1111/nph.16026. 31631362

[msae087-B8] Bateson W, Gregory RP. On the inheritance of heterostylism in *Primula*. Proc R Soc B. 1905:76:581–586.

[msae087-B9] Blischak PD, Barker MS, Gutenkunst RN. Inferring the demographic history of inbred species from genome-wide SNP frequency data. Mol Biol Evol. 2020:37(7):2124 2136. 10.1093/molbev/msaa042. 32068861 PMC7828618

[msae087-B10] Broad Institute . 2019. [accessed 2019 May 02]. http://broadinstitute.github.io/picard/.

[msae087-B11] Brůna T, Hoff KJ, Lomsadze A, Stanke M, Borodovsky M. BRAKER2: automatic eukaryotic genome annotation with GeneMark-EP+ and AUGUSTUS supported by a protein database. NAR Genom Bioinform. 2021:3(1):lqaa108. 10.1093/nargab/lqaa108. 33575650 PMC7787252

[msae087-B12] Burgarella C, Gayral P, Ballenghien M, Bernard A, David P, Jarne P, Correa A, Hurtrez-Boussès S, Escobar J, Galtier N, et al Molecular evolution of freshwater snails with contrasting mating systems. Mol Biol Evol. 2015:32(9):2403–2416. 10.1093/molbev/msv121. 25980005

[msae087-B13] Burgarella C, Glémin S. Population genetics and genome evolution of selfing species. In eLS. Chichester: John Wiley & Sons Ltd; 2017. p. 1–8.

[msae087-B14] Bushnell B . 2015. [accessed 2019 Apr 03]. sourceforge.net/projects/bbmap/.

[msae087-B15] Camacho C, Coulouris G, Avagyan V, Ma N, Papadopoulos J, Bealer K, Madden TL. BLAST+: architecture and applications. BMC Bioinformatics. 2009:10(1):421. 10.1186/1471-2105-10-421. 20003500 PMC2803857

[msae087-B16] Castanera R, Morales-Diaz N, Gupta S, Purugganan M, Casacuberta JM. Transposons are important contributors to gene expression variability under selection in rice populations. eLife. 2023:12:RP86324. 10.7554/eLife.86324.3. 37467142 PMC10393045

[msae087-B17] Chang CC, Chow CC, Tellier LC, Vattikuti S, Purcell SM, Lee JJ. Second-generation PLINK: rising to the challenge of larger and richer datasets. GigaScience. 2015:4(1):7. 10.1186/s13742-015-0047-8. 25722852 PMC4342193

[msae087-B18] Charlesworth B, Charlesworth D. The maintenance and breakdown of distyly. Am Nat. 1979a:114(4):499–513. 10.1086/283497.

[msae087-B19] Charlesworth D, Charlesworth B. A model for the evolution of distyly. Am Nat. 1979b:114(4):467–498. 10.1086/283496.

[msae087-B20] Chen S, Zhou Y, Chen Y, Gu J. fastp: an ultra-fast all-in-one FASTQ preprocessor. Bioinformatics. 2018:34(17):i884–i890. 10.1093/bioinformatics/bty560. 30423086 PMC6129281

[msae087-B21] Cutter AD . Reproductive transitions in plants and animals: selfing syndrome, sexual selection and speciation. New Phyt. 2019:224(3):1080–1094. 10.1111/nph.16075. 31336389

[msae087-B22] Danecek P, Auton A, Abecasis G, Albers CA, Banks E, DePristo MA, Handsaker RE, Lunter G, Marth GT, Sherry ST, et al The variant call format and VCFtools. Bioinformatics. 2011:27(15):2156–2158. 10.1093/bioinformatics/btr330. 21653522 PMC3137218

[msae087-B23] Danecek P, McCarthy SA. BCFtools/csq: haplotype-aware variant consequences. Bioinformatics. 2017:33(13):2037–2039. 10.1093/bioinformatics/btx100. 28205675 PMC5870570

[msae087-B24] Darwin C . On the existence of two forms and on their reciprocal sexual relation in several species of the genus *Linum*. Bot J Linn Soc. 1863:7(26):69–83.

[msae087-B25] Darwin C . The effects of cross and self fertilisation in the vegetable kingdom. Cambridge: Cambridge University Press; Cambridge Core; 1876.

[msae087-B26] Darwin C . The different forms of flowers on plants of the same species. London: John Murray; 1877.

[msae087-B27] Davis MB, Shaw RG. Range shifts and adaptive responses to quaternary climate change. Science. 2001:292(5517):673–679. 10.1126/science.292.5517.673. 11326089

[msae087-B28] Dobin A, Gingeras TR. Mapping RNA-Seq reads with STAR. Curr Protoc Bioinform. 2015:51(1):11.14.1–11.14.19. 10.1002/0471250953.bi1114s51. PMC4631051 26334920

[msae087-B29] Dowrick VPJ . Heterostyly and homostyly in *Primula obconica*. Heredity (Edinb). 1956:10(2):219–236. 10.1038/hdy.1956.19.

[msae087-B30] Dudchenko O, Batra SS, Omer AD, Nyquist SK, Hoeger M, Durand NC, Shamim MS, Machol I, Lander ES, Aiden AP, et al *De novo* assembly of the *Aedes aegypti* genome using Hi-C yields chromosome-length scaffolds. Science. 2017:356(6333):92–95. 10.1126/science.aal3327. 28336562 PMC5635820

[msae087-B31] Edgar RC . MUSCLE: multiple sequence alignment with high accuracy and high throughput. Nucleic Acids Res. 2004:32(5):1792–1797. 10.1093/nar/gkh340. 15034147 PMC390337

[msae087-B32] Ernst A . Heterostylie-Forschung. Versuche zur genetischen Analyse eines Organisations-und “Anpassungs” merkmales. Z induktive Abstammungs Vererbungsl. 1936:71(71):156–230.

[msae087-B33] Evanno G, Regnaut S, Goudet J. Detecting the number of clusters of individuals using the software Structure: a simulation study. Mol Ecol. 2005:14(8):2611–2620. 10.1111/j.1365-294X.2005.02553.x. 15969739

[msae087-B34] Fawcett JA, Takeshima R, Kikuchi S, Yazaki E, Katsube-Tanaka T, Dong Y, Li M, Hunt HV, Jones MK, Lister DL, et al Genome sequencing reveals the genetic architecture of heterostyly and domestication history of common buckwheat. Nat Plants. 2023:9(8):1236–1251. 10.1038/s41477-023-01474-1. 37563460

[msae087-B35] Fitak RR . *Optm*: estimating the optimal number of migration edges on population trees using *Treemix*. Biol Methods Protoc. 2021:6(1):bpab017. 10.1093/biomethods/bpab017. 34595352 PMC8476930

[msae087-B36] Fulton TM, Chunwongse J, Tanksley SD. Microprep protocol for extraction of DNA from tomato and other herbaceous plants. Plant Mol Biol Rep. 1995:13(3):207–209. 10.1007/BF02670897.

[msae087-B37] Gabriel L, Hoff KJ, Brůna T, Borodovsky M, Stanke M. TSEBRA: transcript selector for BRAKER. BMC Bioinformatics. 2021:22(1):566. 10.1186/s12859-021-04482-0. 34823473 PMC8620231

[msae087-B38] Ganders FR . The biology of heterostyly. New Zeal J Bot. 1979:17(4):607–635. 10.1080/0028825X.1979.10432574.

[msae087-B39] Goodstein DM, Shu S, Howson R, Neupane R, Hayes RD, Fazo J, Mitros T, Dirks W, Hellsten U, Putnam N, et al Phytozome: a comparative platform for green plant genomics. Nucleic Acids Res. 2012:40(D1):D1178–D1186. 10.1093/nar/gkr944. 22110026 PMC3245001

[msae087-B40] Groom SV, Stevens MI, Schwarz MP. Parallel responses of bees to Pleistocene climate change in three isolated archipelagos of the southwestern Pacific. Proc R Soc B. 2014:281(1785):20133293. 10.1098/rspb.2013.3293. PMC4024284 24807250

[msae087-B41] Gu Z, Gu L, Eils R, Schlesner M, Brors B. *Circlize* implements and enhances circular visualization in R. Bioinformatics. 2014:30(19):2811–2812. 10.1093/bioinformatics/btu393. 24930139

[msae087-B42] Gutenkunst RN, Hernandez RD, Williamson SH, Bustamante CD. Inferring the joint demographic history of multiple populations from multidimensional SNP frequency data. PLoS Genet. 2009:5(10):e1000695. 10.1371/journal.pgen.1000695. 19851460 PMC2760211

[msae087-B43] Gutiérrez-Valencia J, Fracassetti M, Berdan EL, Bunikis I, Soler L, Dainat J, Kutschera VE, Losvik A, Désamoré A, Hughes PW, et al Genomic analyses of the *Linum* distyly supergene reveal convergent evolution at the molecular level. Curr Biol. 2022a:32(20):4360–4371.e6. 10.1016/j.cub.2022.08.042. 36087578

[msae087-B44] Gutiérrez-Valencia J, Fracassetti M, Horvath R, Laenen B, Désamore A, Drouzas AD, Friberg M, Kolár F, Slotte T. Genomic signatures of sexual selection on pollen-expressed genes in *Arabis alpina*. Mol Biol Evol. 2022b:39(1):msab349. 10.1093/molbev/msab349. 34878144 PMC8788238

[msae087-B45] Gutiérrez-Valencia J, Hughes PW, Berdan EL, Slotte T. The genomic architecture and evolutionary fates of supergenes. Genome Biol Evol. 2021:13(5):evab057. 10.1093/gbe/evab057. 33739390 PMC8160319

[msae087-B46] Hartfield M, Bataillon T, Glémin S. The evolutionary interplay between adaptation and self-fertilization. Trends Genet. 2017:33(6):420–431. 10.1016/j.tig.2017.04.002. 28495267 PMC5450926

[msae087-B47] Henning PM, Shore JS, McCubbin AG. The *S*-gene *YUC6* pleiotropically determines male mating type and pollen size in heterostylous *Turnera* (Passifloraceae): a novel neofunctionalization of the YUCCA gene family. Plants. 2022:11(19):2640. 10.3390/plants11192640. 36235506 PMC9572539

[msae087-B48] Hoff KJ, Lange S, Lomsadze A, Borodovsky M, Stanke M. BRAKER1: unsupervised RNA-Seq-based genome annotation with GeneMark-ET and AUGUSTUS. Bioinformatics. 2016:32(5):767–769. 10.1093/bioinformatics/btv661. 26559507 PMC6078167

[msae087-B49] Huu CN, Kappel C, Keller B, Sicard A, Takebayashi Y, Breuninger H, Nowak MD, Bäurle I, Himmelbach A, Burkart M, et al Presence versus absence of *CYP734A50* underlies the style-length dimorphism in primroses. eLife. 2016:5:e17956. 10.7554/eLife.17956. 27596932 PMC5012859

[msae087-B50] Huu CN, Plaschil S, Himmelbach A, Kappel C, Lenhard M. Female self-incompatibility type in heterostylous *Primula* is determined by the brassinosteroid-inactivating cytochrome P450 CYP734A50. Curr Biol. 2022:32(3):671–676.e5. 10.1016/j.cub.2021.11.046. 34906354

[msae087-B51] Innes PA, Smart BC, Barham JAM, Hulke BS, Kane NC. 2023. Chromosome-scale genome assembly of lewis flax (*Linum lewisii* Pursh.), unpublished data, bioRxiv 2023.10.10.561607. 10.1101/2023.10.10.561607.

[msae087-B52] Johnson SD, Dafni A. Response of bee-flies to the shape and pattern of model flowers: implications for floral evolution in a Mediterranean herb. Funct Ecol. 1998:12(2):289–297. 10.1046/j.1365-2435.1998.00175.x.

[msae087-B53] Keightley PD, Jackson BC. Inferring the probability of the derived vs. the ancestral allelic state at a polymorphic site. Genetics. 2018:209(3):897–906. 10.1534/genetics.118.301120. 29769282 PMC6028244

[msae087-B54] Korunes KL, Samuk K. Pixy: unbiased estimation of nucleotide diversity and divergence in the presence of missing data. Mol Ecol Resour. 2021:21(4):1359–1368. 10.1111/1755-0998.13326. 33453139 PMC8044049

[msae087-B55] Kumar S, Stecher G, Li M, Knyaz C, Tamura K. MEGA x: molecular evolutionary genetics analysis across computing platforms. Mol Biol Evol. 2018:35(6):1547–1549. 10.1093/molbev/msy096. 29722887 PMC5967553

[msae087-B56] Kurtz S, Phillippy A, Delcher AL, Smoot M, Shumway M, Antonescu C, Salzberg SL. Versatile and open software for comparing large genomes. Genome Biol. 2004:5(2):R12. 10.1186/gb-2004-5-2-r12. 14759262 PMC395750

[msae087-B57] Laenen B, Tedder A, Nowak MD, Toräng P, Wunder J, Wötzel S, Steige KA, Kourmpetis Y, Odong T, Drouzas AD, et al Demography and mating system shape the genome-wide impact of purifying selection in *Arabis alpina*. Proc Natl Acad Sci U S A. 2018:115(4):816–821. 10.1073/pnas.1707492115. 29301967 PMC5789905

[msae087-B58] Laibach F . Die abweichungen vom ‘‘mechanischen’’ zahlenverhältnis der long- under kurz-griffel bei heterostylen pflanzen. Biol Zentralbl. 1923:43:148–157.

[msae087-B59] Larsson A . AliView: a fast and lightweight alignment viewer and editor for large datasets. Bioinformatics. 2014:30(22):3276–3278. 10.1093/bioinformatics/btu531. 25095880 PMC4221126

[msae087-B60] Levin DA . Mating system shifts on the trailing edge. Ann Botany. 2012:109(3):613–620. 10.1093/aob/mcr159. 21980190 PMC3278285

[msae087-B61] Li H . 2013. Aligning sequence reads clone sequences and assembly contigs with BWA-MEM, arXiv, arXiv:1303.3997, preprint: not peer reviewed.

[msae087-B62] Li H . Minimap2: pairwise alignment for nucleotide sequences. Bioinformatics. 2018:34(18):3094–3100. 10.1093/bioinformatics/bty191. 29750242 PMC6137996

[msae087-B63] Li J, Cocker JM, Wright J, Webster MA, McMullan M, Dyer S, Swarbreck D, Caccamo M, van Oosterhout C, Gilmartin PM. Genetic architecture and evolution of the *S* locus supergene in *Primula vulgaris*. Nat Plants. 2016:2(12):16188. 10.1038/nplants.2016.188. 27909301

[msae087-B64] Lloyd DG, Webb CJ. The evolution of heterostyly. In: Barrett SCH, editor. Evolution and function of heterostyly. Berlin: Springer-Verlag; 1992. p. 151–178.

[msae087-B65] Love MI, Huber W, Anders S. Moderated estimation of fold change and dispersion for RNA-Seq data with DESeq2. Genome Biol. 2014:15(12):550. 10.1186/s13059-014-0550-8. 25516281 PMC4302049

[msae087-B66] Löytynoja A, Goldman N. webPRANK: a phylogeny-aware multiple sequence aligner with interactive alignment browser. BMC Bioinformatics. 2010:11(1):579. 10.1186/1471-2105-11-579. 21110866 PMC3009689

[msae087-B67] Maguilla E, Escudero M, Ruz-Martín J, Arroyo J. Origin and diversification of flax and their relationship with heterostyly across the range. J Biogeogr. 2021:48(8):1994–2007. 10.1111/jbi.14129.

[msae087-B68] Manni M, Berkeley MR, Seppey M, Zdobnov EM. BUSCO: assessing genomic data quality and beyond. Curr Protoc. 2021:1(12):e323. 10.1002/cpz1.323. 34936221

[msae087-B69] Mather K, De Winton D. Adaptation and counter-adaptation of the breeding system in Primula: the nature of breeding systems. Ann Bot. 1941:5(2):297–311. 10.1093/oxfordjournals.aob.a087394.

[msae087-B70] Mattila TM, Laenen B, Slotte T. Population genomics of transitions to selfing in Brassicaceae model systems. In: Dutheil JY, editor. Statistical population genomics. New York (NY): Springer; 2020. p. 269–287. 10.1007/978-1-0716-0199-0_11 31975171

[msae087-B71] McDill J, Repplinger M, Simpson BB, Kadereit JW. The phylogeny of *Linum* and Linaceae subfamily Linoideae with implications for their systematics biogeography and evolution of heterostyly. Syst Bot. 2009:34(2):386–405. 10.1600/036364409788606244.

[msae087-B72] Messer PW, Petrov DA. Frequent adaptation and the McDonald-Kreitman test. Proc Natl Acad Sci U S A. 2013:110(21):8615–8620. 10.1073/pnas.1220835110. 23650353 PMC3666677

[msae087-B73] Mora-Carrera E, Stubbs RL, Keller B, Léveillé-Bourret É, de Vos JM, Szövényi P, Conti E. Different molecular changes underlie the same phenotypic transition: origins and consequences of independent shifts to homostyly within species. Mol Ecol. 2023:32(1):61–78. 10.1111/mec.16270. 34761469 PMC10078681

[msae087-B74] Mori T, Kuroiwa H, Higashiyama T, Kuroiwa T. GENERATIVE CELL SPECIFIC 1 is essential for angiosperm fertilization. Nat Cell Biol. 2006:8(1):64–71. 10.1038/ncb1345. 16378100

[msae087-B75] Murga-Moreno J, Coronado-Zamora M, Hervas S, Casillas S, Barbadilla A. iMKT: the integrative McDonald and Kreitman test. Nucleic Acids Res. 2019:47(W1):W283–W288. 10.1093/nar/gkz372. 31081014 PMC6602517

[msae087-B76] Murray BG . Floral biology and self-incompatibility in *Linum*. Bot Gazette. 1986:147(3):327–333. 10.1086/337599.

[msae087-B77] Naiki A . Heterostyly and the possibility of its breakdown by polyploidization. Plant Species Biol. 2012:27(1):3–29. 10.1111/j.1442-1984.2011.00363.x.

[msae087-B78] Nei M, Gojobori T. Simple methods for estimating the numbers of synonymous and nonsynonymous nucleotide substitutions. Mol Biol Evol. 1986:3(5):418–426. 10.1093/oxfordjournals.molbev.a040410. 3444411

[msae087-B79] Orueta D . Thermal relationships between *Calendula arvensis* inflorescences and *Usia aurata* bombyliid flies. Ecology. 2002:83(11):3073–3085. 10.1890/0012-9658(2002)083[3073:TRBCAI]2.0.CO;2.

[msae087-B80] Ossowski S, Schneeberger K, Lucas-Lledó JI, Warthmann N, Clark RM, Shaw RG, Weigel D, Lynch M. The rate and molecular spectrum of spontaneous mutations in *Arabidopsis thaliana*. Science. 2010:327(5961):92–94. 10.1126/science.1180677. 20044577 PMC3878865

[msae087-B81] Page JT, Liechty ZS, Huynh MD, Udall JA. BamBam: genome sequence analysis tools for biologists. BMC Res Notes. 2014:7(1):829. 10.1186/1756-0500-7-829. 25421351 PMC4258253

[msae087-B82] Paradis E . Pegas: an R package for population genetics with an integrated-modular approach. Bioinformatics. 2010:26(3):419–420. 10.1093/bioinformatics/btp696. 20080509

[msae087-B83] Pastor J, Diosdado JC, Santa Bárbara C, Vioque J, Pérez E. Números cromosómicos para la flora Española: 556-619. Lagascalia. 1990:15:271–274.

[msae087-B84] Pérez-Barrales R, Armbruster WS. Incomplete partitioning of pollinators by *Linum suffruticosum* and its coflowering congeners. Am J Bot. 2023:110(6):e16181. 10.1002/ajb2.16181. 37163619

[msae087-B85] Pertea M, Pertea GM, Antonescu CM, Chang T-C, Mendell JT, Salzberg SL. StringTie enables improved reconstruction of a transcriptome from RNA-Seq reads. Nat Biotechnol. 2015:33(3):290–295. 10.1038/nbt.3122. 25690850 PMC4643835

[msae087-B86] Pickrell JK, Pritchard JK. Inference of population splits and mixtures from genome-wide allele frequency data. PLoS Genet. 2012:8(11):e1002967. 10.1371/journal.pgen.1002967. 23166502 PMC3499260

[msae087-B87] Piper JG, Charlesworth B, Charlesworth D. Breeding system evolution in *Primula vulgaris* and the role of reproductive assurance. Heredity (Edinb). 1986:56(2):207–217. 10.1038/hdy.1986.33.

[msae087-B88] POWO . Plants of the World Online. Facilitated by the Royal Botanic Gardens Kew. 2024. [accessed 2024 Jan 24]. http://www.plantsoftheworldonline.org/.

[msae087-B89] Quinlan AR, Hall IM. BEDTools: a flexible suite of utilities for comparing genomic features. Bioinformatics. 2010:26(6):841–842. 10.1093/bioinformatics/btq033. 20110278 PMC2832824

[msae087-B90] R Core Team . 2021. R: A language and environment for statistical computing. R Foundation for Statistical Computing. https://www.R-project.org/.

[msae087-B91] Repplinger M . 2009. Phylogenie und Biogeographie der Linoideae (Linaceae) – Evolution von Heterostylie/Homostylie in Linum [PhD thesis]. Mainz (Germany): Johannes Gutenberg-Universität.

[msae087-B92] Roach MJ, Schmidt SA, Borneman AR. Purge Haplotigs: allelic contig reassignment for third-gen diploid genome assemblies. BMC Bioinformatics. 2018:19(1):460. 10.1186/s12859-018-2485-7. 30497373 PMC6267036

[msae087-B93] Ruiz-Martín J, Santos-Gally R, Escudero M, Midgley JJ, Pérez-Barrales R, Arroyo J. Style polymorphism in *Linum* (Linaceae): a case of Mediterranean parallel evolution? Plant Biol. 2018:20(S1):100–111. 10.1111/plb.12670. 29164751

[msae087-B94] Schiestl FP, Johnson SD. Pollinator-mediated evolution of floral signals. Trends Ecol Evol. 2013:28(5):307–315. 10.1016/j.tree.2013.01.019. 23480953

[msae087-B95] Sendrowski J, Bataillon T. fastDFE: fast and flexible inference of the distribution of fitness effects. Mol Biol Evol. 2024. 10.1093/molbev/msae070. PMC11140822 38577958

[msae087-B96] Shimizu KK, Tsuchimatsu T. Evolution of selfing: recurrent patterns in molecular adaptation. Annu Rev Ecol Evol S. 2015:46(1):593–622. 10.1146/annurev-ecolsys-112414-054249.

[msae087-B97] Shore JS, Hamam HJ, Chafe PDJ, Labonne JDJ, Henning PM, McCubbin AG. The long and short of the *S*-locus in *Turnera* (Passifloraceae). New Phyt. 2019:224(3):1316–1329. 10.1111/nph.15970. 31144315

[msae087-B98] Simón-Porcar VI, Escudero M, Santos-Gally R, Sauquet H, Schönenberger J, Johnson SD, Arroyo J. Convergent evolutionary patterns of heterostyly across angiosperms support the pollination-precision hypothesis. Nat Comm. 2024:15(1):1237. 10.1038/s41467-024-45118-0. PMC10858259 38336937

[msae087-B99] Slotte T . The impact of linked selection on plant genomic variation. Brief Funct Genomics. 2014:13(4):268–275. 10.1093/bfgp/elu009. 24759704 PMC4110415

[msae087-B100] Slotte T, Foxe JP, Hazzouri KM, Wright SI. Genome-wide evidence for efficient positive and purifying selection in *Capsella grandiflora*, a plant species with a large effective population size. Mol Biol Evol. 2010:27(8):1813–1821. 10.1093/molbev/msq062. 20194429

[msae087-B101] Slotte T, Hazzouri KM, Ågren JA, Koenig D, Maumus F, Guo Y-L, Steige K, Platts AE, Escobar JS, Newman LK, et al The *Capsella rubella* genome and the genomic consequences of rapid mating system evolution. Nat Genet. 2013:45(7):831–835. 10.1038/ng.2669. 23749190

[msae087-B102] Smit AFA, Hubley R, Green P. 2013. RepeatMasker (v.4.1.0). https://www.repeatmasker.org/.

[msae087-B103] Smit AFA, Hubleyo R. 2008. RepeatModeler. https://www.repeatmasker.org/RepeatModeler/.

[msae087-B104] Song B, Marco-Sola S, Moreto M, Johnson L, Buckler ES, Stitzer MC. AnchorWave: sensitive alignment of genomes with high sequence diversity, extensive structural polymorphism, and whole-genome duplication. Proc Natl Acad Sci U S A. 2022:119(1):e2113075119. 10.1073/pnas.2113075119. PMC8740769 34934012

[msae087-B105] Stanke M, Diekhans M, Baertsch R, Haussler D. Using native and syntenically mapped cDNA alignments to improve *de novo* gene finding. Bioinformatics. 2008:24(5):637–644. 10.1093/bioinformatics/btn013. 18218656

[msae087-B106] Stebbins GL . Self-fertilization and population variability in the higher plants. Am Nat. 1957:91(861):337–354. 10.1086/281999.

[msae087-B107] Stecher G, Tamura K, Kumar S. Molecular Evolutionary Genetics Analysis (MEGA) for macOS. Mol Biol Evol. 2020:37(4):1237–1239. 10.1093/molbev/msz312. 31904846 PMC7086165

[msae087-B108] Steige KA, Reimegård J, Koenig D, Scofield DG, Slotte T. *Cis*-regulatory changes associated with a recent mating system shift and floral adaptation in *Capsella*. Mol Biol Evol. 2015:32(10):2501–2514. 10.1093/molbev/msv169. 26318184 PMC4576713

[msae087-B109] Tataru P, Mollion M, Glémin S, Bataillon T. Inference of distribution of fitness effects and proportion of adaptive substitutions from polymorphism data. Genetics. 2017:207(3):1103–1119. 10.1534/genetics.117.300323. 28951530 PMC5676230

[msae087-B110] Thorvaldsdóttir H, Robinson JT, Mesirov JP. Integrative Genomics Viewer (IGV): high-performance genomics data visualization and exploration. Brief Bioinform. 2013:14(2):178–192. 10.1093/bib/bbs017. 22517427 PMC3603213

[msae087-B111] Tsuchimatsu T, Goubet PM, Gallina S, Holl A-C, Fobis-Loisy I, Bergès H, Marande W, Prat E, Meng D, Long Q. Patterns of polymorphism at the self-incompatibility locus in 1083 *Arabidopsis thaliana* genomes. Mol Biol Evol. 2017:34(8):1878–1889. 10.1093/molbev/msx122. 28379456 PMC5850868

[msae087-B112] Ushijima K, Nakano R, Bando M, Shigezane Y, Ikeda K, Namba Y, Kume S, Kitabata T, Mori H, Kubo Y. Isolation of the floral morph-related genes in heterostylous flax *Linum grandiflorum*: the genetic polymorphism and the transcriptional and post-transcriptional regulations of the *S* locus. Plant J. 2012:69(2):317–331. 10.1111/j.1365-313X.2011.04792.x. 21923744

[msae087-B113] Valdés-Florido A, Tan L, Maguilla E, Simón-Porcar VI, Zhou Y-H, Arroyo J, Escudero M. Drivers of diversification in *Linum* (Linaceae) by means of chromosome evolution: correlations with biogeography breeding system and habit. Ann Bot. 2023:132(5):949–962. 10.1093/aob/mcad139. 37738171 PMC10808019

[msae087-B114] Walker BJ, Abeel T, Shea T, Priest M, Abouelliel A, Sakthikumar S, Cuomo CA, Zeng Q, Wortman J, Young SK, et al Pilon: an integrated tool for comprehensive microbial variant detection and genome assembly improvement. PLoS One. 2014:9(11):e112963. 10.1371/journal.pone.0112963. 25409509 PMC4237348

[msae087-B115] Wang X-J, Barrett SCH, Zhong L, Wu Z-K, Li D-Z, Wang H, Zhou W. The genomic selfing syndrome accompanies the evolutionary breakdown of heterostyly. Mol Biol Evol. 2021:38(1):168–180. 10.1093/molbev/msaa199. 32761213 PMC7782863

[msae087-B116] Weir BS, Cockerham CC. Estimating *F*-statistics for the analysis of population structure. Evolution. 1984:38(6):1358–1370. 10.1111/j.1558-5646.1984.tb05657.x. 28563791

[msae087-B117] Williamson RJ, Josephs EB, Platts AE, Hazzouri KM, Haudry A, Blanchette M, Wright SI. Evidence for widespread positive and negative selection in coding and conserved noncoding regions of *Capsella grandiflora*. PLoS Genet. 2014:10(9):e1004622. 10.1371/journal.pgen.1004622. 25255320 PMC4178662

[msae087-B118] Wright S . The genetical structure of populations. Ann Eugen. 1949:15(1):323–354. 10.1111/j.1469-1809.1949.tb02451.x. 24540312

[msae087-B119] Wright S . Evolution and the genetics of populations. In: Volume 2: The theory of gene frequencies. Chicago: The University of Chicago Press; 1969.

[msae087-B120] Wright SI, Kalisz S, Slotte T. Evolutionary consequences of self-fertilization in plants. Proc R Soc B. 2013:280(1760):20130133. 10.1098/rspb.2013.0133. PMC3652455 23595268

[msae087-B121] Yang J, Xue H, Li Z, Zhang Y, Shi T, He X, Barrett SCH, Wang Q, Chen J. Haplotype-resolved genome assembly provides insights into the evolution of *S*-locus supergene in distylous *Nymphoides indica*. New Phyt. 2023:240(5):2058–2071. 10.1111/nph.19264. 37717220

[msae087-B122] Yasui Y, Mori M, Aii J, Abe T, Matsumoto D, Sato S, Hayashi Y, Ohnishi O, Ota T. *S-LOCUS EARLY FLOWERING 3* is exclusively present in the genomes of short-styled buckwheat plants that exhibit heteromorphic self-incompatibility. PLoS One. 2012:7(2):e31264. 10.1371/journal.pone.0031264. 22312442 PMC3270035

[msae087-B123] Yi H, Wang J, Wang J, Rausher M, Kang M. Genomic insights into inter- and intraspecific mating system shifts in *Primulina*. Mol Ecol. 2022:31(22):5699–5713. 10.1111/mec.16706. 36178058

[msae087-B124] Yuan S, Barrett SCH, Duan T, Qian X, Shi M, Zhang D. Ecological correlates and genetic consequences of evolutionary transitions from distyly to homostyly. Ann Bot. 2017:120(5):775–789. 10.1093/aob/mcx098. 28961784 PMC5691548

[msae087-B125] Zhao Z, Zhang Y, Shi M, Liu Z, Xu Y, Luo Z, Yuan S, Tu T, Sun Z, Zhang D, et al Genomic evidence supports the genetic convergence of a supergene controlling the distylous floral syndrome. New Phyt. 2023:237(2):601–614. 10.1111/nph.18540. 36239093

[msae087-B126] Zhong L, Barrett SCH, Wang X-J, Wu Z-K, Sun H-Y, Li D-Z, Wang H, Zhou W. Phylogenomic analysis reveals multiple evolutionary origins of selfing from outcrossing in a lineage of heterostylous plants. New Phyt. 2019:224(3):1290–1303. 10.1111/nph.15905. 31077611

