# Supplementary material for: Genetic Causes and Genomic Consequences of Breakdown of Distyly in Linum trigynum
Source: Mol Biol Evol. 2024 May 6;41(5):msae087. doi: 10.1093/molbev/msae087 (PMC11114476; doi:10.1093/molbev/msae087)
Supplement: msae087_Supplementary_Data [file msae087_supplementary_data.pdf]

Genetic causes and genomic consequences of breakdown of distyly in *Linum trigynum*

Supplementary Information

Juanita Gutiérrez-Valencia[1], Panagiotis-Ioannis Zervakis[1], Zoé Postel[1], Marco Fracassetti[1], Aleksandra Losvik[1], Sara Mehrabi[1], Ignas Bunikis[2], Lucile Soler[3], P. William Hughes[1], Aurélie Désamoré[1], Benjamin Laenen[1], Mohamed Abdelaziz[4], Olga Vinnere Pettersson[2], Juan Arroyo[5], Tanja Slotte[1,*]

[1]Department of Ecology, Environment and Plant Sciences, Science for Life Laboratory, Stockholm University, Stockholm, Sweden
[2]Uppsala Genome Center, Department of Immunology, Genetics and Pathology, Uppsala University, Uppsala, Sweden
[3]Department of Medical Biochemistry and Microbiology, Uppsala University, National Bioinformatics Infrastructure Sweden (NBIS), Science for Life Laboratory, Uppsala University, Uppsala, Sweden
[4]Department of Genetics, University of Granada, Granada, Spain
[5]Department of Plant Biology and Ecology, University of Seville, Seville, Spain

*Corresponding author:
Tanja Slotte, Dept. of Ecology, Environment and Plant Sciences, Stockholm University, Stockholm SE-106 91 Stockholm, Sweden. Email: tanja.slotte@su.se

Supplementary Methods

Plant Material

For genome sequencing and population genomic analyses, mature fruits and leaves of *L. trigynum* and *L. tenue* were sampled in 16 different localities of southern Spain, representing eight populations per species (Table S1, Supplementary Material). The material was collected in individual bags per mother plant and dried with silica gel for 24 to 48 hours. Seeds were sterilized with a wash of 10% bleach solution with liquid detergent, followed by rinsing with 70% ethanol and sterile, distilled water. Sterilized seeds were located in Murashige-Skoog medium (Sigma Aldrich, USA) and covered with a thin layer of top agar, followed by 25 days of stratification at 4°C. For germination, seeds in petri dishes were located in controlled climate chamber at Stockholm University (Stockholm, Sweden) set to 16 h light at 20°C: 8 h dark at 18°C, 60% maximum humidity, 122 µE light intensity. After eight days in the climate chamber, seedlings were transplanted to pots containing a mixture of equal parts of vermiculite and perlite for two parts of regular soil (Hasselfors Garden, Sweden). Leaves from one individual per seed family were taken with sterilized forceps, placed in 2 mL Eppendorf tubes, and flash frozen in liquid nitrogen. For expression analyses, floral buds and leaves sampled as described in Gutiérrez-Valencia et al. (2022a). Samples were used for different sequencing experiments as explained in *DNA extraction and sequencing*.

DNA and RNA extraction and sequencing

To produce whole-genome sequencing data for population genomic studies, genomic DNA from young frozen leaves was extracted using the kit Isolate II Plant DNA (Bioline, UK). DNA concentration and purity were measured using Qubit and NanoDrop respectively. Sequencing libraries were prepared from 1µg of DNA using TruSeq PCRfree DNA sample preparation kit from Illumina (cat# 20015962) targeting an insert size of 350 bp, following the manufacturers' instructions. Sequencing was conducted with a NovaSeq 6000 system from Illumina using a S4 flowcell (150-bp paired-end reads, v1.5 sequencing chemistry). Library preparation and sequencing were executed at the SNP&SEQ Technology Platform at the National Genomics Infrastructure in Uppsala (Sweden).

Three individuals of *L. trigynum* were randomly chosen to generate the data that would be further used for the production of *de novo* genome assemblies. To produce HiFi PacBio reads from sample *Ltri*. 6-30-2 (39.3606, -3.9477; Castilla de La Mancha, Spain) high Molecular Weight (HMW) DNA was extracted from young leaves using a protocol that involved a first extraction using the CTAB method (as in Fulton et al., 1995), followed by two purification steps with the kit Genomic tip 500/G (QIAGEN, Germany). DNA concentration was measured with Qubit, and DNA purity was quantified with NanoDrop. Sample integrity and fragment size distribution were examined with a pulsed-field capillary electrophoresis using a Femto Pulse system. DNA was sequenced on 2 Sequel II SMRT cells in HiFi mode, which led to the production of 27 Gb of HiFi data. Library preparation and sequencing were conducted at the Uppsala Genome Center from the National Genomics Infrastructure (NGI), Uppsala, Sweden. For sequencing with the 10X Chromium protocol for linked-reads, HMW DNA from two samples of *L. trigynum* (*Ltri.* 1-1-1 and *Ltri.* 1-42-2) from Castilla La Mancha, Spain, and four samples of *L. tenue* (*Lten*. CL-3-1, *Lten*. CL-75-1, *Lten*. STM-5-2, *Lten.* STM 30-1) from two natural populations in Castillo de Locubín, Spain (CL) and Santa María de Trassiera, Spain (STM), respectively, and one sample of *L. maritimum* was extracted using the kit Genomic-tip 100/G (QIAGEN, Germany). Sequencing libraries were prepared from 0.6 ng of DNA using the Chromium Genome Library preparation kit (cat# 120~260/58/61/62) following the manufacturers' protocol (#CG00043 Chromium Genome Reagent Kit v2 User Guide). The library was sequenced on a HiSeqX system (paired-end 150bp read length, v2.5 sequencing chemistry) at the SNP&SEQ Technology Platform at the National Genomics Infrastructure in Uppsala (Sweden).

To assist the scaffolding of the HiFi PacBio reads, we generated Dovetail Hi-C data from the same individual from which we obtained long reads (*Ltri*. 6-30-2). Young leaves were frozen with liquid nitrogen and ground. The sample was prepared for cross-linking, followed by cell lysis and proximity-ligation. The library was prepared according to manufacturer's instructions, followed by sequencing on a NovaSeq6000 system (NovaSeq Control Software 1.6.0/RTA v3.4.4, setup = 2x151 setup, workflow= NovaSeqXp, flowcell mode = S4). Sample and library preparation, and library

sequencing were conducted at the Genomics Applications platform at the National Genomics Infrastructure in Stockholm, Sweden.

To annotate the genome assembly, we generated RNA sequencing (RNAseq) data from two technical replicates of four types of samples (stems, leaves, floral buds, and mature flowers) from the same individual used for long-read genome assembly. For differential expression analyses, we further extracted RNA from floral buds and leaves of *L. tenue* and *L. trigynum* (Sample size: *L. tenue* thrum=6, pin=4, *L. trigynum* homostyle=5). Total RNA was extracted using the RNEasy Plant Mini Kit (QIAGEN, Germany) following the manufacturer's instructions. We evaluated RNA quantity and quality using an Agilent Bioanalyzer 2100 (Agilent Technologies, Santa Clara, USA) with RNA Plant Nano microfluidic chips. RNAseq libraries were generated using the TruSeq stranded mRNA library preparation kit (Illumina, San Diego) including polyA selection. Libraries were sequenced on an Illumina NovaSeq S1 Sequencing System to produce paired-end 150bp read length sequences using the v1 chemistry at the SNP&SEQ Technology Platform in Uppsala (Sweden).

Reannotation of LtTSS1

We reannotated *LtTSS1* in *L. tenue* to improve on the automated annotation and fully consider RNA-seq evidence (see Methods for details). This was necessary as the original annotation was based on Augustus predictions without including RNA-seq evidence. While the exon-intron structure in our original annotation of *LtWDR-44* waas supported by RNA-seq evidence, we found that for *LtTSS1* the exon-intron structure originally predicted was not well supported by the RNA-seq data (Figure S3a-b).

We conducted additional PCR-based validation of our new annotation of *LtTSS1*. Specifically, we conducted a series of PCR reactions with primer pairs complementary to the template within the first exon in our new annotation and towards the 3'UTR (Table S4). As a template for the PCR reactions we used both the cDNA obtained from mRNA extracted from *L. tenue* thrum flower buds with RNeasy Plant Mini kit (Qiagen) and reverse-transcribed using SuperScript IV First-Strand Synthesis System (Invitrogen), as well as DNA extracted from *L. tenue* thrum leaves (Quick-DNA MagBead Plus kit, Zymo research). PCR was performed with MyTaq DNA Polymerase (Meridian Bioscience) using a program with initial denaturation at 95°C for 3 min, followed by 30 cycles of 95°C for 2s, 55°C for 2s and 72°C for 3s, and final extension at 72°C for 1 min. Reaction products were separated on 2% agarose gel and stained with GelRed (Biotium).

We found that, in contrast to expectations given the exon-intron structure in the original annotation, PCR products amplified from cDNA and genomic DNA were the same length for all primer pairs located before primer R6 (Figure S3c-d), supporting our new annotation of *LtTSS1*.

References

Fulton, T. M., Chunwongse, J., & Tanksley, S. D. (1995). Microprep protocol for extraction of DNA from tomato and other herbaceous plants. *Plant Molecular Biology Reporter*, *13*, 207–209.

Gutiérrez-Valencia, J., Fracassetti, M., Berdan, E. L., Bunikis, I., Soler, L., Dainat, J., Kutschera, V. E., Losvik, A., Désamoré, A., Hughes, P. W., Foroozani, A., Laenen, B., Pesquet, E., Abdelaziz, M., Pettersson, O. V., Nystedt, B., Brennan, A. C., Arroyo, J., & Slotte, T. (2022a). Genomic analyses of the *Linum* distyly supergene reveal convergent evolution at the molecular level. *Current Biology*, *32*, 4360-4371.

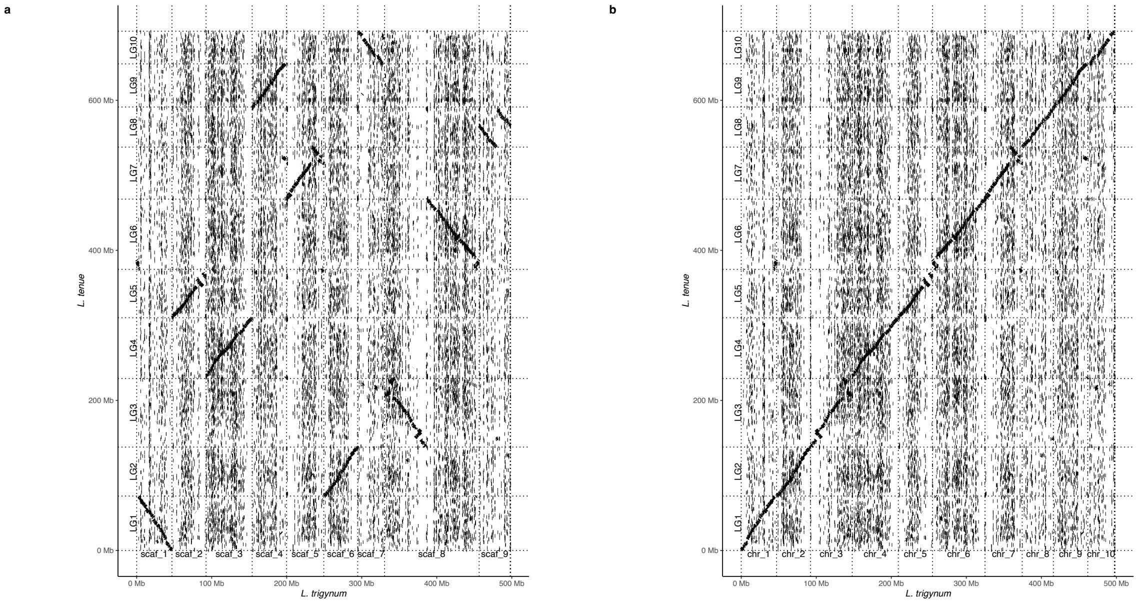

Fig. S1. Synteny between *L. tenue* and *L. trigynum* assemblies. (a) Alignment between the *L. tenue* genome assembly and the scaffolded *L. trigynum* assembly (b) Alignment between the *L. tenue* genome and the final *L. trigynum* assembly. Both the alignments were produced using minimap2 v2.4, and visualized with pafr (https://github.com/dwinter/pafr).

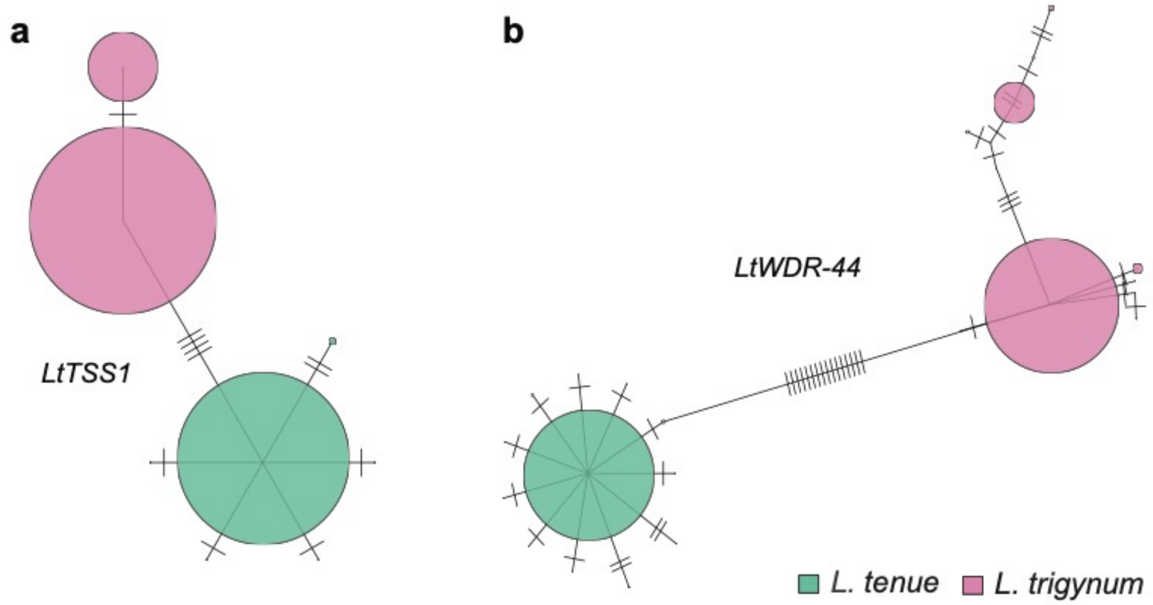

Fig. S2. Haplotype networks for *LtTSS1* and *LtWDR-44* based on population genomic data. (a) Haplotype network of *LtTSS1*. (b) Haplotype network of *LtWDR-44*. For both genes, the *L. trigynum S*-locus haplotype is connected to only one haplotype of *L. tenue*, consistent with a single *S*-haplotype from the distylous ancestor contributing to genetic variation at the *L. trigynum S*-locus. The size of each circle is proportional to the number of chromosomes harboring each haplotype.

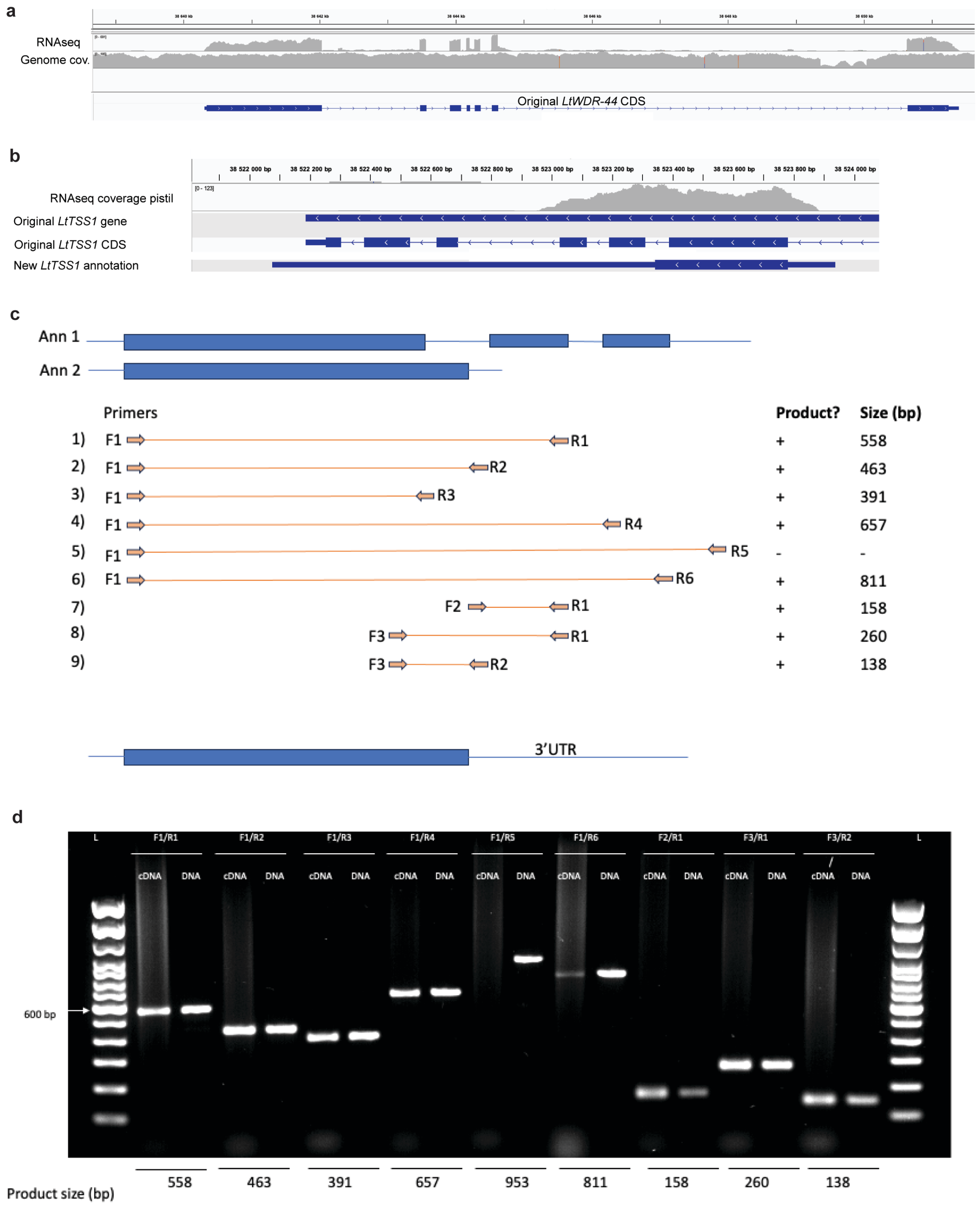

Fig. S3. Reannotation and validation. (a) The original annotation of *LtWDR-44* was well supported by RNA-seq data. (b) The original annotation (annotation 1) of *LtTSS1* was not well supported by RNA-seq coverage, as coverage only extends to the first three exons, and the exon-intron structure does not seem supported. (b) Locations of PCR primers in relation to exons 1-3 of the original annotation (Ann 1) and exon 1 of our new annotation of *LtTSS1* (Ann 2). The supported gene model of *LtTSS1* is shown at the bottom. (c) PCR results of selected primer pairs using as a template cDNA synthesized based on mRNA from flower buds and DNA extracted from leaves of *L. tenue* thrum individuals. DNA ladder (L) = 100bp DNA ladder, Thermo Fisher Scientific. These assays support our new annotation of *LtTSS1* with one exon, and delimit the length of the 3'UTR (based on reaction 5 which yielded no amplification from a cDNA template in contrast to amplification from genomic DNA which was successful).

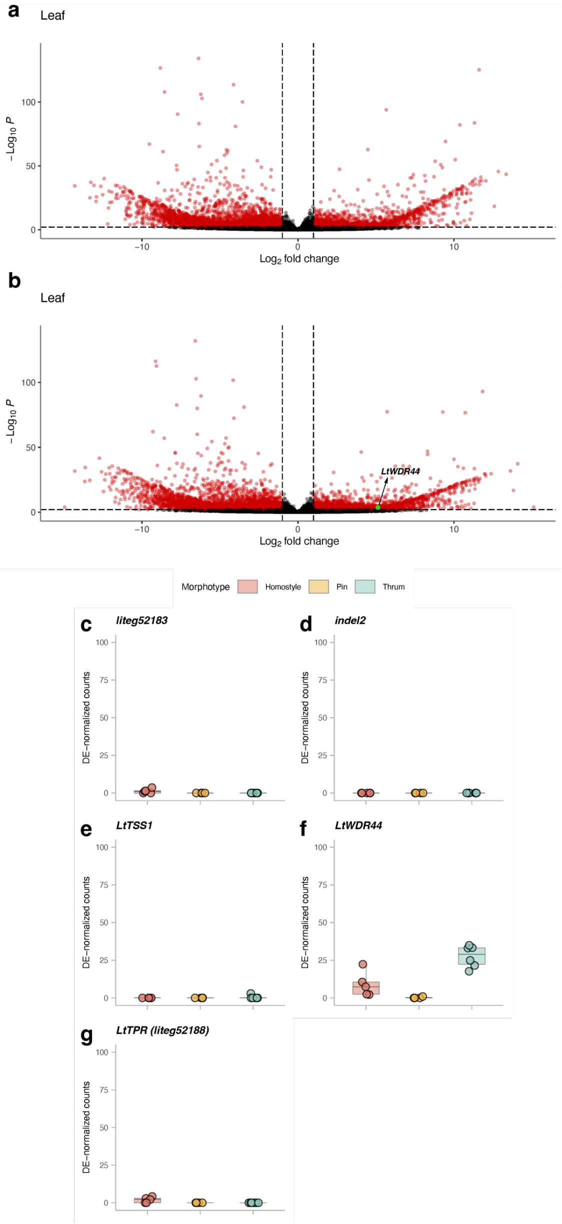

Fig. S4. Differential expression of *S*-linked genes in leaves. (a) Volcano plot showing fold change vs significance of differential expression between *L. trigynum* (homostyle) and *L. tenue* thrum. (b) Volcano plot showing fold change vs significance of differential expression between *L. trigynum* (homostyle) and *L. tenue* pin. Candidate genes with significant differential expression are marked in the plot. (c-e) Expression levels of *S*-linked candidate genes in *L. trigynum*, *L. tenue* pin and thrum individuals.

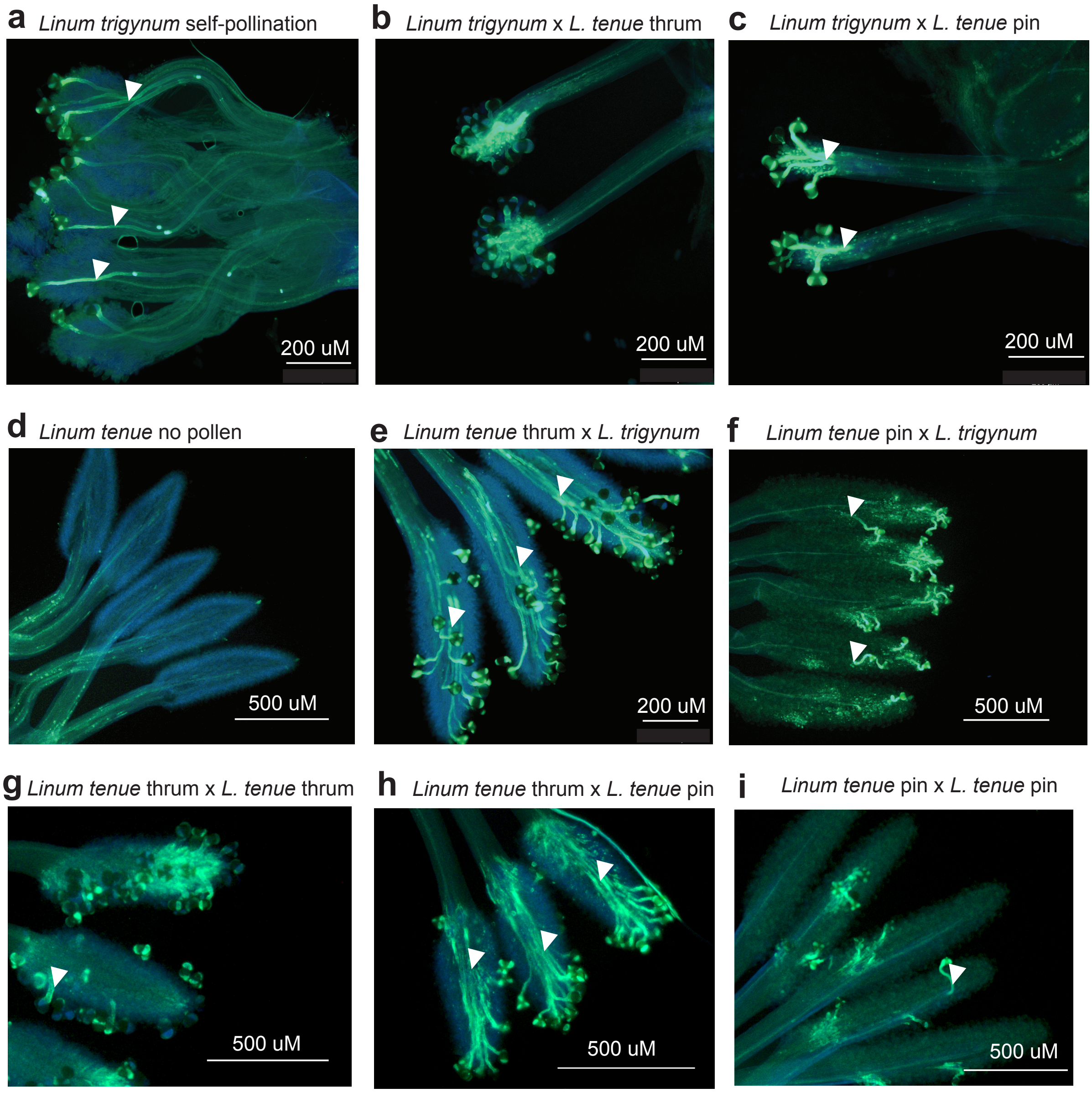

Fig. S5. Pollination assays indicate altered male SI function in *L. trigynum*. Fluorescence micrographs depicting pollen tube growth in styles after controlled crosses. Panels a) and d) show positive and negative controls, respectively. Panels b) and c) show weak or no pollen tube growth of *L. tenue* pollen on *L. trigynum* stigmas. Panels e) and f) show that *L. trigynum* pollen has full pollen tube growth on *L. tenue* thrum but not pin stigmas. Panels g) and i) depict incompatible *L. tenue* crosses, and panel h) a compatible *L. tenue* cross. Pollen tube growth of *L. trigynum* pollen on *L. tenue* thrum stigmas (e) resembles the outcome of a compatible *L. tenue* x *L. trigynum* cross (h). Scale bars indicate the degree of magnification, and arrows indicate pollen tubes. Note that the site of rejection in incompatible *Linum* crosses is in the style.

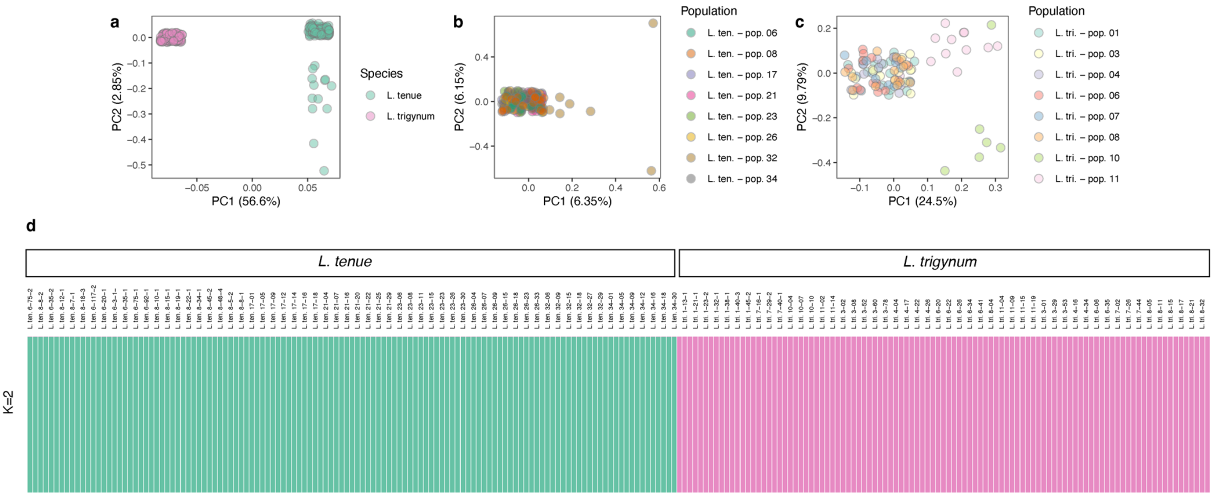

Fig. S6. Principal component analyses of genome-wide non-coding SNPs. Principal component analyses of 76,934 SNPs indicate that (a) *L. tenue* and *L. trigynum* are clearly separated along the first principal component (explaining 56.6% of variation), and populations of *L. tenue* cluster more closely together (b) than those of *L. trigynum* (c), consistent with weaker population structure in *L. tenue* than *L. trigynum*. (d) When only *K*=2 are considered, population structure analyses with ADMIXTURE place all *L. tenue* individuals and all *L. trigynum* individuals in two separate clusters.

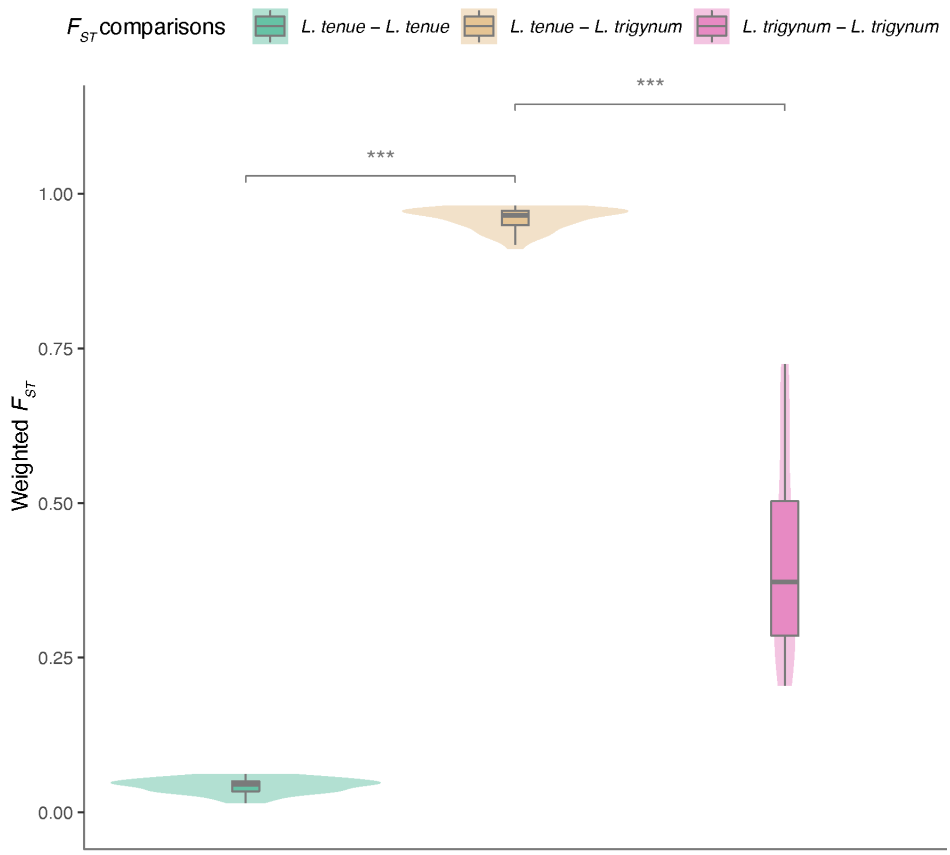

Fig. S7. Comparisons of F_{ST} estimates within *L. tenue* and *L. trigynum*, and between populations of both species. F_{ST} estimates between *L. tenue* and *L. trigynum* populations were the highest (median=0.96, 1st and 3st quantiles=0.95 and 0.97), followed by estimates within *L. trigynum* (median=0.37, 1st and 3st quantile=0.28 and 0.50), and the lowest F_{ST} values were obtained for *L. tenue* populations (median=0.05, 1st and 3st quantile=0.03 and 0.05). ***P < 0.001 Kruskal–Wallis test followed by Dunn's test after Bonferroni correction.

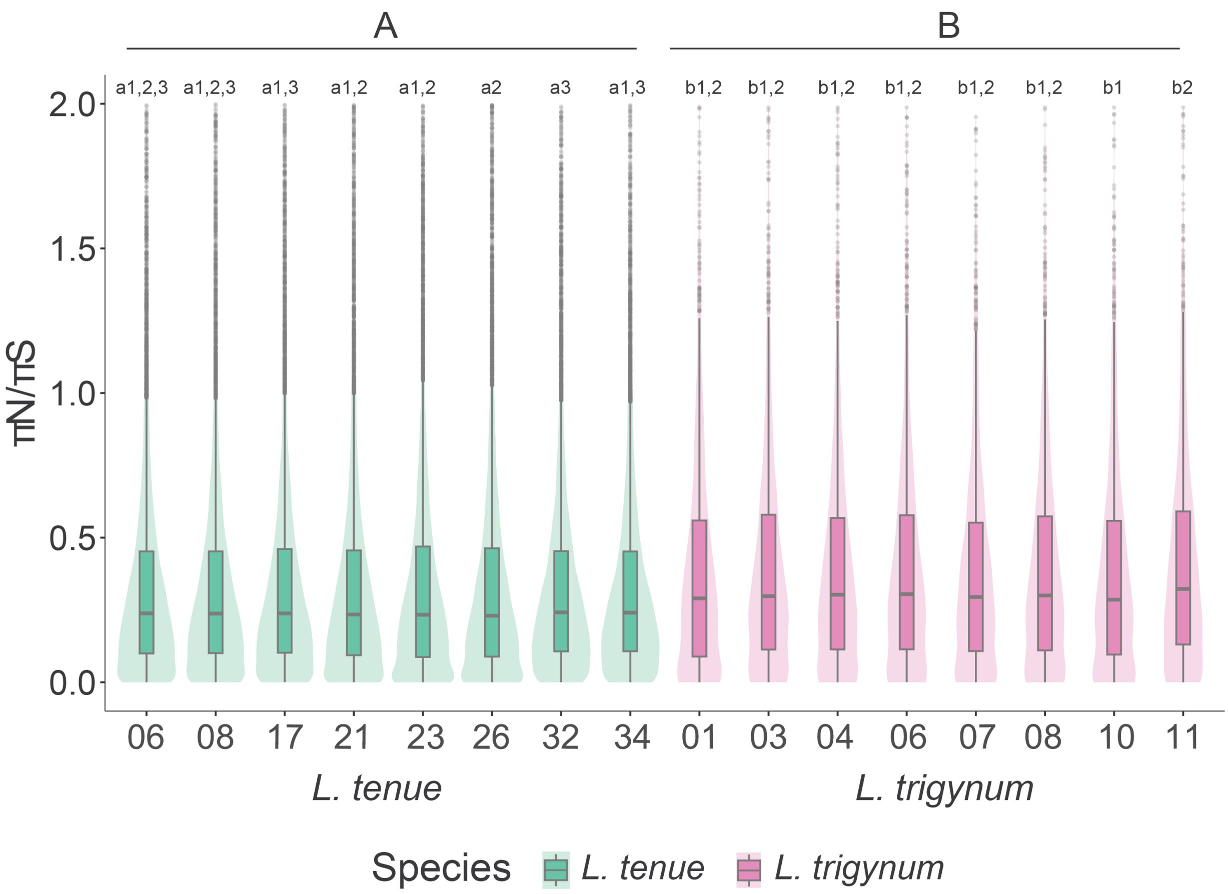

Fig. S8. Distribution of ratios of nonsynonymous to synonymous polymorphism in populations of *L. tenue* and *L. trigynum*. Different letters denote statistically significant π_N / π_S estimates between populations and between species; Kruskal–Wallis test followed by Dunn's test with Benjamini-Hochberg corrected *P* values, $P < 0.05$. π_N/π_S estimates for *L. tenue* and *L. trigynum* populations. Comparisons of π_N / π_S estimates show that *L. tenue* (A, mean=0.40, S.E.=0.02, n=8) exhibits a significantly higher ratio of nonsynonymous to synonymous nucleotide polymorphism compared to *L. trigynum* (B, mean=0.50, S.E.=0.15, n=8).

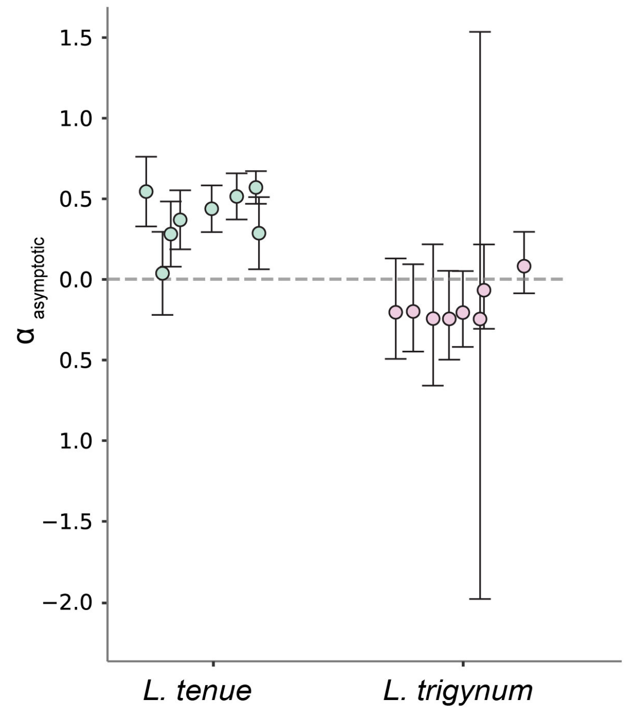

Fig. S9. Comparison of the contribution of positive selection to nonsynonymous divergence in *L. tenue* and *L. trigynum*. The plot shows point estimates of alpha, estimated using Messer and Petrov's (2013) asymptotic method, along with 95% confidence intervals (CIs) estimated assuming a Poisson distribution of deleterious fixations. Note that estimation of alpha in population *L. trigynum Ltri.*10 (with the widest CI) triggered a warning due to low numbers of polymorphic sites.

Table S1. List of populations. Populations represented in the data set used to investigate the genomic consequences of mating system shift in *L. trigynum*. The number of individuals sequenced is indicated by n. The Source column gives the study accession number at the European Nucleotide Archive (https://www.ebi.ac.uk/ena/).

Species and Population	Population identifier	n	Latitude	Longitude	Locality	Province	Country	Source
L. tenue - pop. 17	*L. ten.* 17	14	37.0286	-5.6084	El Coronil, Sevilla	Andalusia	Spain	ENA: PRJEB67577
L. tenue - pop. 21	*L. ten.* 21	14	36.9238	-5.3960	La Muela, Cádiz	Andalusia	Spain	ENA: PRJEB67577
L. tenue - pop. 23	*L. ten.* 23	14	36.7923	-4.9920	El Burgo, Málaga	Andalusia	Spain	ENA: PRJEB67577
L. tenue - pop. 26	*L. ten.* 26	14	37.9053	-6.6166	Los Marines, Huelva	Andalusia	Spain	ENA: PRJEB67577
L. tenue - pop. 32	*L. ten.* 32	13	36.4637	-5.4898	Jimena de la Frontera, Cádiz	Andalusia	Spain	ENA: PRJEB67577
L. tenue - pop. 34	*L. ten.* 34	13	36.7486	-5.6552	Arcos de la Frontera, Cádiz	Andalusia	Spain	ENA: PRJEB67577
L. tenue - pop. 06	*L. ten.* 06	26	37.5640	-3.8897	Castillo de Locubín, Valdepeñas de Jaén, Jaén	Andalusia	Spain	ENA: PRJEB52918
L. tenue - pop. 08	*L. ten.* 08	17	37.9320	-4.8833	Santa María de Trassierra, Sierra Morena	Córdoba	Spain	ENA: PRJEB52918
L. trigynum - pop. 01	*L. tri.* 01	16	38.4008	-4.2775	Fuencaliente, Ciudad Real	Castilla de La Mancha	Spain	ENA: PRJEB67577
L. trigynum - pop. 10	*L. tri.* 10	6	37.8816	-6.6190	Linares de la Sierra, Huelva	Andalusia	Spain	ENA: PRJEB67577
L. trigynum - pop. 11	*L. tri.* 11	12	37.9090	-6.7314	Jabugo, Huelva	Andalusia	Spain	ENA: PRJEB67577
L. trigynum - pop. 03	*L. tri.* 03	14	38.4864	-4.3357	Cam. de Ventillas, Fuencaliente, Ciudad Real	Castilla de La Mancha	Spain	ENA: PRJEB67577
L. trigynum - pop. 04	*L. tri.* 04	13	39.1280	-3.9347	Fernán Caballero, Ciudad Real	Castilla de La Mancha	Spain	ENA: PRJEB67577
L. trigynum - pop. 06	*L. tri.* 06	14	39.3606	-3.9477	Los Yébenes, Toledo	Castilla de La Mancha	Spain	ENA: PRJEB67577
L. trigynum - pop. 07	*L. tri.* 07	12	39.5816	-3.9287	Marjaliza, Toledo	Castilla de La Mancha	Spain	ENA: PRJEB67577
L. trigynum - pop. 08	*L. tri.* 08	14	38.5314	-4.0951	Mestanza, Ciudad Real	Castilla de La Mancha	Spain	ENA: PRJEB67577

Table S2. Assembly statistics for the *L. trigynum* genome assembly. These statistics concern the assembly based on PacBio HiFi and OmniC data.

Assembly step	Primary assembly (Mbp)	No. sequences	N50 (Mbp)	L50	BUSCO score (eukaryota_odb10)
IPA	498.1	103	12.6	12	C: 96.0% [S: 66.0%, D:30%], F: 0.9%, M:3.0%.
IPA + 3D-DNA scaffolding with OmniC data	498.1	13	47.0	4	C:95.6%[S:66.2%,D:29.4%],F:1.2%,M:3.2%
Final assembly	498.1	15	47.0	5	C:95.6%[S:66.2%,D:29.4%],F:1.2%,M:3.2%

Table S3. Assembly statistics for Supernova assemblies. These statistics concern 10x linked-read read draft assemblies.

Name[1]	ENA[2]	p10[3]	m10[4]	scaffold_N50[5]	L50_tot[6]	min_scaf_L50_tot[7]	effective_coverage_median[8]	assembly_size[9]	est_genome_size[10]	Hetdist[11]
Lten_CL-3-1	this study	59.8529	15.8589	122358	819	130000	32.4453	522480488	669217000	231
Lten_CL-75-1	this study	130.5	15.5328	173115	543	190000	32.9326	531458269	650952000	247
Lten_STM-5-2	this study	60.2059	15.5481	111376	975	120000	37.2382	534978380	639943000	300
Lten_STM-18-3	GCA_946150905, GCA_946150875	178.088	16.7389	157491	557	170000	28.0475	514042692	643490000	228
Lten_STM-21-2	GCA_946151035.1, GCA_946151015.1	42.8235	23.5384	80702	1073	90000	24.1663	437801162	650493000	212
Lten_STM-30-1	this study	127.235	16.5916	268883	245	3,00E+05	36.2522	473121657	647544000	223
Lten_STM-50-1	GCA_946150985, GCA_946151025	37.9412	17.7777	123569	758	140000	29.1046	503721094	645600000	217
Ltri_1-1-1	this study	78.2647	3.19013	4821513	33	4860000	49.5904	461688410	653639000	11164
Ltri_1-42-2	this study	77.4706	3.66056	4468862	32	4460000	60.7739	463460708	648321000	8009
Lmar-08	this study	15.3235	35.0141	33037	5977	29503	13.4518	601440318	1288340000	4575

[1]Name used in this study.
[2]ENA accession numbers of the two pseudohaplotypes.
[3]p10: for an average point on the genome, the estimated number of molecules that extend 10kb in both directions from that point, counting both alleles.
[4]m10: the estimated percent of genomic kmers that are either missing from the assembly entirely or present only in scaffolds shorter than 10 kb.
[5]scaffold_N50: the N50 size of the compressed scaffolds.
[6]L50_tot: number of scaffolds containing half of the genome length.
[7]min_scaf_L50_tot: length of the smallest L50 scaffold.
[8]effective_coverage: estimated effective coverage. This is the estimated deduplicated coverage of an average base on the genome, counting both alleles.
[9]assembly_size: the number of bases in the compressed scaffolds.
[10]est_genome_size: estimated genome size in bases, computed from the distribution of kmers.
[11]hetdist: mean distance in bases between heterozygous sites.

Table S4. Sequences of PCR primers for validation of *LtTSS1* cDNA structure. Primer abbreviations correspond to Fig. S3.

Primer abbreviation	Primer name	Primer sequence
F1	Lten_TSS1_ex1_F1	TAAGTCTGTGACCGGCAAGC
F2	TSS1_FW2	TTTCCCCGATTCTACTTCCA
F3	Lten_TSS1_ex1_F2	GAGGGGATTGAAGATACGGCT
R1	Lten_Junction_1_R1	TCCACGAGAGGGTCAGTAGG
R2	Lten_TSS1_int1_R	AGCGTTGGATGTTCAGTGGA
R3	Lten_Junction_2_R1	GTCGTTCCTAACCATGGAACTGT
R4	Lten_TSS1_int2_ex3_R	TTGATCCTTCATTTCCATGCCTC
R5	Lten_TSS1_3UTR_R1	CCTGGCGAATTGTGTGACCT
R6	Lten_TSS1_3UTR_R2	AAGGAATCGGTATATCGAATGGCA

Table S5. Repeat annotation in the promoter region 2 kb upstream of the transcription start site of the two *S*-locus genes *LtWDR-44* and *LtTSS1*on pseudochromosome 10 in *L. tenue* and *L. trigynum*.

Gene	Species	Start position	End position	Classification
LtWDR-44	*L. trigynum*	30612087	30612173	LTR/Ty3
		30612113	30612174	Unknown
		30612602	30613000	LTR/Ty3
		30613084	30613226	Unknown
		30613262	30613418	Unknown
		30613503	30613564	DNA/hAT-Tip100
		30613568	30613722	LINE/L1
	L. tenue	38638631	38638778	Unknown
		38638779	38639068	DNA/MULE-MuDR
		38639564	38639857	DNA/MULE-MuDR
		38639844	38640021	Unknown
		38640008	38640176	LINE/L1
LtTSS1	*L. trigynum*	30534704	30534759	LTR
		30534776	30535157	LTR
		30535398	30536104	DNA/hAT-Ac
		30536271	30538731	LTR/Copia
	L. tenue	38523985	38524034	LTR
		38524015	38524084	LTR
		38524100	38524139	LTR
		38524109	38524467	LTR/Copia
		38524693	38525413	DNA/hAT-Ac
		38525574	38526300	LTR/Copia
